# Allelic Variation in Maize *Malate Dehydrogenase 7* Shapes Promoter Methylation and Banded Leaf and Sheath Blight Resistance

**DOI:** 10.1002/advs.202511356

**Published:** 2025-11-20

**Authors:** Luyang Wei, Junbin Chen, Chuang Liu, DanDan Liu, Meida Du, Shengfeng He, Wenyu Cheng, Vijai Bhadauria, You‐Liang Peng, Wangsheng Zhu

**Affiliations:** ^1^ State Key Laboratory of Maize Bio‐breeding Key Laboratory of Surveillance and Management for Plant Quarantine Pests of Ministry of Agriculture and Rural Affairs College of Plant Protection China Agricultural University Beijing 100193 P. R. China; ^2^ College of Plant Protection China Agricultural University Beijing 100193 P. R. China

**Keywords:** banded leaf and sheath blight, DNA methylation, maize, malate dehydrogenase, natural variation, WRKY transcriptional factor 44

## Abstract

*Rhizoctonia solani*, a globally distributed phytopathogen, causes banded leaf and sheath blight (BLSB) in maize and sheath blight in rice, severely threatening crop productivity. Here, this work identifies *ZmRRS1* (Resistance to *Rhizoctonia solani* 1), which encodes maize malate dehydrogenase, as a key positive regulator of BLSB resistance in the field through a genome‐wide association study. *ZmRRS1* knockout lines exhibit increased susceptibility to BLSB, whereas overexpression of *ZmRRS1* and its homologs confer enhanced resistance to *R. solani* in both maize and rice. Using heterozygote inbred families (HIF), this work uncovers that the presence and absence of a 831‐bp transposable element (TE) element is the causal polymorphism that determines the differential expression pattern of the two *ZmRRS1* alleles in response to *R. solani*. Furthermore, the absence of the TE increases the binding of the ZmWRKY44 transcription factor to the W‐box motif in the promoter of *ZmRRS1*, thereby enhancing BLSB resistance. Transcriptomic analysis reveals that *ZmRRS1* potentiates reactive oxygen species (ROS)‐mediated immunity against BLSB. Notably, the overexpression of *ZmRRS1* does not incur agronomic penalties under normal growth conditions. Collectively, this work identifies *ZmRRS1* as a positive regulator of BLSB resistance, offering valuable genetic resources for breeding BLSB‐resistant maize and rice varieties.

## Graphical abstract

In this study maize chloroplastic *malate dehydrogenase7* (*ZmMDH7*), is identified as a *Rhizoctonia solani* resistance gene in maize. *ZmMDH7* is regulated by transcription factor ZmWRKY44 via pathogens challenge to elevate mitochondrial ROS and SA signaling pathway. A transposable element insertion in promotor of *ZmRRS1* dampens ZmWRKY44‐mediated expression of *ZmMDH7*, that suppress maize resistance to *R. solani*.

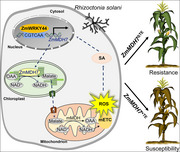

## 1 Introduction

Maize (*Zea mays* L.), a crucial crop for food, feed, and fuel, faces challenges from extreme weather conditions and diseases. Banded leaf and sheath blight (BLSB), caused by the soil‑borne fungus *Rhizoctonia solani* remains a global production constraint, and is ubiquitous across the major maize‑growing regions of Asia, the Americas, and Africa.^[^ ^1^ ^]^ The same pathogen causes rice sheath blight, one of the top three diseases that limit the yield of cultivated rice (*Oryza sativa*) globally.^[^ ^2^ ^]^ BLSB routinely reduces yields by 10–40% and may lead to total crop failure.^[^ ^3^ ^]^ Extensive sheath necrosis often spreads to the ears and husks, ultimately killing the plant and causing severe economic losses. Thus, deploying durable resistance is crucial for achieving sustainable maize production and securing food security.

*R. solani* resistance is a quantitative trait governed by multiple loci.^[^ ^4^ ^]^ Over the past decades, quantitative trait loci (QTLs) mapping for *R. solani* have uncovered several resistance intervals in maize and rice.^[^ ^5^, ^6^, ^7^, ^8^ ^]^ Despite the availability of maize germplasm with partial resistance to *R. solani*, a fully immune cultivar has yet to be reported.^[^ ^8^ ^]^ Genome‐wide association study (GWAS), transcriptome profiling, and allied approaches have identified several maize sheath‑blight quantitative resistance genes (e.g., *ZmFBL41*, *ZmNCED6*, *ZmPUB19*) providing valuable gene resources for breeding BLSB‐resistant varieties.^[^ ^9^, ^10^, ^11^ ^]^ In rice, decades of genetic analysis have largely yielded minor‐effect QTL, with functional evidence accumulating for canonical defense modules, WRKY factors, polygalacturonase inhibitors, chitinases, and ROS‐linked regulators.^[^ ^4^, ^8^, ^12^, ^13^ ^]^ Recently, optimized inoculation methods uncovered *OsSBRR1*, a G‐type lectin receptor‐like kinase, is a key locus for sheath blight resistance in rice; natural allelic variation (*SBRR1*‐R) delivers substantial phenotypic gains and clear breeding utility.^[^ ^14^ ^]^ Across wheat and soybean, *Rhizoctonia* resistance is still QTL‐only, with no positional cloning yet (e.g., Qse.xn‐2BL; soybean root‐rot QTL).^[^ ^15^, ^16^ ^]^ Together, these observations reveal a broader gap in our understanding of *R. solani* resistance across cereals and legumes, underscoring the need for high‐resolution dissection of natural variation in crops.

Reactive oxygen species (ROS) play a fundamental role in plant innate immunity by orchestrating multi‐layered defense responses.^[^ ^17^ ^]^ An NADPH oxidase‐mediated apoplastic ROS burst initiates signaling cascades, including calcium influx, MAP kinase activation, and transcriptional reprogramming.^[^ ^17^ ^]^ The subcellular origin of ROS dictates downstream defense responses: chloroplast‐derived ROS act as retrograde signals that link photosynthesis to systemic acquired resistance; mitochondrial ROS pulses trigger programmed cell death (PCD) and enhance resistance independently of plasma‐membrane NADPH oxidases; and peroxisomal H_2_O_2_ integrates cytosolic signaling with hormonal pathway.^[^ ^18^, ^19^, ^20^, ^21^ ^]^ Extensive studies in model and crop species, including *Arabidopsis*, rice, and maize, have established ROS signaling as a central component of plant disease resistance and highlight its potential for improving maize resistance to BLSB.^[^ ^22^, ^23^, ^24^ ^]^

Malate dehydrogenases (MDHs), NAD⁺/NADP⁺‑dependent oxidoreductases that interconvert malate and oxaloacetate, serve as redox hubs across multiple organelles.^[^ ^25^ ^]^ MDHs have been extensively studied in the field of abiotic stress, including salt, drought, cold, nutrient deficiency, and heavy‐metal toxicity.^[^ ^25^, ^26^, ^27^, ^28^, ^29^ ^]^ Emerging evidence also implicates MDHs in immune signaling: In cassava, MeMDH1 dimerization boosts malate synthesis and elevates salicylic acid and *PR1* expression, enhancing disease resistance.^[^ ^30^ ^]^ In *Arabidopsis*, plastidial plNAD‑MDH and mitochondrial mNAD‑MDH1 form a malate shuttle that reallocates reducing equivalents to trigger mitochondrial ROS‑mediated PCD.^[^ ^31^ ^]^ Beyond stress responses, a legume‐specific SnRK1‐MDH module was recently shown to promote cytosolic malate biosynthesis, ensuring carbon supply to bacteroids and supporting symbiotic nitrogen fixation.^[^ ^32^ ^]^ Collectively, MDHs function as redox rheostats that reprogram defense signaling and metabolic processes while also facilitating carbon supply for symbiotic nitrogen fixation. However, mechanistic insights into MDH‐mediated immune regulation remain fragmented, and crop‐resistance MDH alleles remain largely unexplored.

Transposable elements (TEs) pose a potential threat to genome integrity, prompting eukaryotes to silence these elements via epigenetic mechanisms such as DNA methylation.^[^ ^33^ ^]^ DNA methylation, the covalent addition of a methyl group to cytosine, is a conserved epigenetic mark essential for regulating gene expression.^[^ ^34^ ^]^ RNA‐directed DNA methylation (RdDM) enforces transcriptional gene silencing at transposons, whereas small interfering RNAs drive post‐transcriptional decay of homologous transcripts.^[^ ^34^ ^]^ DNA methylation restricts transcription mainly through two intertwined mechanisms: directly by obstructing transcription factor binding or indirectly through methyl‐CpG‐binding proteins, which recruit repressive chromatin‐modifying complexes.^[^ ^35^, ^36^, ^37^ ^]^ TEs, comprising ≈85% of the maize genome, serve as primary targets for DNA methylation, frequently propagating methylation into adjacent promoter regions, condensing chromatin structure, and blocking transcription‐factor recruitment.^[^ ^38^, ^39^, ^40^ ^]^ Such TE‐directed methylation reprograms stress‐ and immunity‐responsive genes, reshaping growth and defense traits.^[^ ^38^, ^39^, ^40^ ^]^

Here, we performed a GWAS on 302 maize inbred lines and identified *ZmRRS1*, encoding a malate dehydrogenase, as a positive regulator of BLSB resistance. In the resistant allele, loss of the 831‐bp TE alleviates TE‐mediated promoter methylation, thereby enhancing ZmWRKY44 binding, boosting pathogen‐induced *ZmRRS1* expression, and strengthening resistance to *R. solani*. Transcriptomic profiling reveals that *ZmRRS1* enhances ROS accumulation and confers resistance to BLSB without compromising agronomic benefits. Thus, the resistant *ZmRRS1* allele represents a valuable genetic resource for breeding *R. solani*‐resistant maize and rice varieties.

## 2 Results

### 2.1 GWAS of BLSB Resistance in Maize

**Figure 1:**
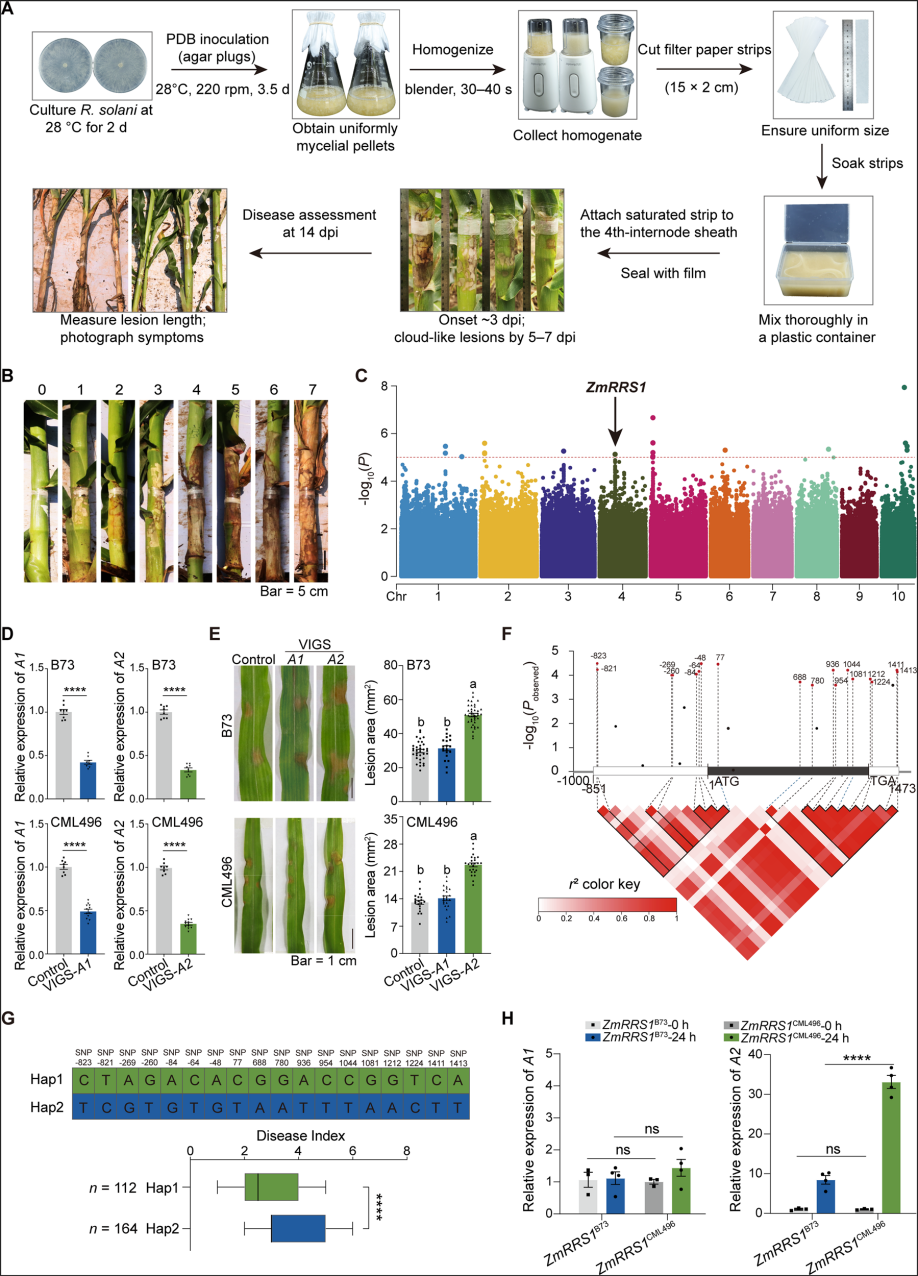
*ZmRRS1* is significantly associated with maize resistance to *R. solani*. A) Workflow for field inoculation of maize leaf sheaths with *R. solani*. Uniform 15 × 2 cm filter‐paper strips were fully saturated with a homogenized *R. solani* potato‐dextrose‐broth (PDB) culture and snugly wrapped around the leaf sheath at the fourth internode of 55‐day‐old (V10) maize plants. The inoculation site was immediately sealed with several layers of cling film to maintain high humidity. Disease progression was assessed 14 dpi: lesions were photographed, the length of lesions recorded, and a disease severity index calculated. B) Representative disease index (DI) grades 0–7 scored 14 days post‐inoculation (dpi) with *R. solani* using the high‐throughput sheath‐wrap method. C) Manhattan plot of the GWAS in 302 maize inbred lines. The dashed horizontal line indicates the Bonferroni‐adjusted significance threshold (*p* = 1 × 10^−5^). A BLSB‐associated locus encompassing *ZmRRS1* was detected on chromosome 4; the lead SNP, chr4.S_83 388 487 (MaizeGDB v2, *p* = 7.48 × 10^−6^), is marked by a black arrow. D,E) Detached‐leaf assays following VIGS‐mediated knockdown of candidate genes *A1* and *A2* in the susceptible maize inbred B73 and the resistant inbred CML496. D) Silencing efficiency assessed by RT‐qPCR at 7 days after CMV infiltration in B73 (top) and CML496 (bottom). Plants infiltrated with the empty vector pCMV201‐2b_N81_‐GFP served as controls. Data represent *n* = 8–12 leaves. E) At 14 days post‐silencing (V2), second true leaves were inoculated with *R. solani* and evaluated at 36 hpi; representative phenotypes (left) and lesion‐area quantification (right) for B73 (top) and CML496 (bottom). Scale bar, 1 cm. Data means ± s.e.m. (*n* = 10–19). Different letters denote significant differences (one‐way ANOVA followed by Tukey's test, *p* < 0.05). F) Linkage‐disequilibrium (LD) heatmap and pairwise LD matrix for 26 SNPs within *ZmRRS1* promoter and coding‐region across 302 maize accessions. The 18 major SNPs are highlighted in red. SNPs in strong LD with the lead SNP form LD blocks, denoted by solid lines and inverted triangles. G) Box‐and‐whisker plot of DI for 112 Hap1 (resistant) and 164 Hap2 (susceptible) inbred lines (haplotypes defined by 18 SNPs). H) Expression of two candidate genes (*A1*‐*A2*) analyzed by RT‐qPCR at 24 h post‐inoculation (hpi) in the leaf sheaths of resistant CML496 (Hap1) and susceptible B73 (Hap2); *A2* (*ZmRRS1*) showed strong induction, with a greater fold‐change in CML496 (means ± s.e.m., *n* = 3–4; two‐way ANOVA with Tukey's test; ns, not significant; **** *p* < 0.0001). In (D) and (G), data are presented as means ± s.e.m.; statistical significance was determined by two‐tailed Student's *t*‐test (****p < 0.0001).

Several inoculation methods for *R. solani* have been reported over recent decades, including seed inoculation with fermented cultures and leaf‐sheath inoculation using fungal blocks or matchsticks.^[^ ^3^, ^9^, ^41^, ^42^, ^43^ ^]^ However, conventional methods often lack consistency in inoculum density, leading to variable disease expression and complicating phenotypic assessments. In this study, we established a simple protocol that delivers a defined, homogeneously mixed inoculum and reproducible symptom development (**Figure** **1**A). The key benefits of this protocol include: 1) consistency and reproducibility, uniform filter‐paper strips are impregnated with a homogeneously mixed fungal suspension, applied to an equivalent leaf‐sheath position, and sealed with plastic wrap, reducing inter‐replicate variance; 2) operational simplicity, streamlined handling minimizes dose and placement variability, enabling reliable, side‐by‐side comparisons across genotypes without specialized equipment.

We used the newly developed protocol to inoculate *R. solani* with a diverse population of naturally varying maize, including 302 distinct inbred lines (≈1500 individuals) under field conditions (Table S1, Supporting Information). This population includes inbred lines of tropical, subtropical, temperate, and mixed origin.^[^ ^44^ ^]^ The disease phenotypes within this population exhibited a normal distribution with disease index (DI) range from 0 to 7, indicating a quantitative feature of BLSB resistance ( 1B; Figure S1A, Supporting Information). Among these, B73 and CML496 represent the susceptible (DI with 7) and resistant (DI with 0) inbred lines, respectively (Figure 1B; Figure S1B, Supporting Information). To identify loci associated with BLSB resistance, a GWAS was conducted using 1252883 SNPs with a minor allele frequency (MAF) ≥ 0.05, covering the entire maize genome.^[^ ^45^ ^]^ The mixed linear model (MLM), which takes population structure into account, identified 23 significant SNPs that exceeded the genome‐wide significance threshold (*P* < 1 × 10^−5^; Figure 1C; Figure S1C, Supporting Information). These significant SNPs correspond to 10 loci on chromosomes 1–5 and 10, with candidate genes listed in Table S2, Supporting Information.

### 2.2 *ZmRRS1* is Significantly Associated with BLSB Resistance

One of the ten significant SNPs, chr4.S_83388487 (MaizeGDB V2; *p* = 7.48 × 10^−6^), maps to chromosome 4 and resides in an intergenic region devoid of any annotated gene in the B73 reference genome (Table S2, Supporting Information). A 130‐kb region flanking this SNP was designated for screening candidate genes. Linkage disequilibrium (LD) analysis of the 260‐kb region revealed an LD block within the downstream 130‐kb interval of the lead SNP (Figure S1D, Supporting Information). Functional annotation within this LD block, based on B73‐REFERENCE‐NAM‐5.0, identified two high‐confidence candidate genes, referred to as *A1* (*GRMZM2G471876*) and *A2* (*GRMZM2G068455*) (Figure S1D, Supporting Information).

We employed the ZMBJ‐CMV VIGS tool to silence these two genes individually in the representative inbred lines B73 and CML496, followed by inoculation with *R. solani* on detached leaves.^[^ ^46^ ^]^ The results revealed that silencing *A2* resulted in a significantly larger lesion area, whereas silencing *A1* showed no noticeable effect compared to the controls, suggesting that *A2* is the candidate within this mapping interval (Figure 1D,E).

Gene *A2*, predicted to encode a malate dehydrogenase (MDH), is designated as *ZmRRS1* (*Resistance to Rhizoctonia solani 1*). Analysis of 18 major SNPs within the promoter and coding region of *ZmRRS1* form the LD block, delineating two major haplotypes across 276 maize inbred lines (Figure 1F; Table S3, Supporting Information). Among these, 112 inbred lines belong to the resistant haplotype (Hap1, mean DI of 2.79), while 164 belong to the susceptible haplotype (Hap2, mean DI of 3.63) (Figure 1G). The highly resistant inbred line CML496 encodes the Hap1 allele (*ZmRRS1* ^CML496^), whereas the highly susceptible B73 harbors the Hap2 allele (*ZmRRS1* ^B73^). We then examined the transcriptional responses of the two candidate genes mentioned above at 24 h post‐inoculation (hpi) with *R. solani* in CML496 and B73. While *A1* showed no significant transcriptional changes, *A2* (*ZmRRS1*) was markedly upregulated in response to *R. solani* infection, with greater induction observed in *ZmRRS1* ^CML496^ compared to *ZmRRS1* ^B73^ (Figure 1H), further supporting the notion that *ZmRRS1* is the candidate gene associated with BLSB resistance within the mapping interval.

### 2.3 *ZmRRS1* and its Homologs Positively Regulate *R. Solani* Resistance in Maize and Rice

**Figure 2:**
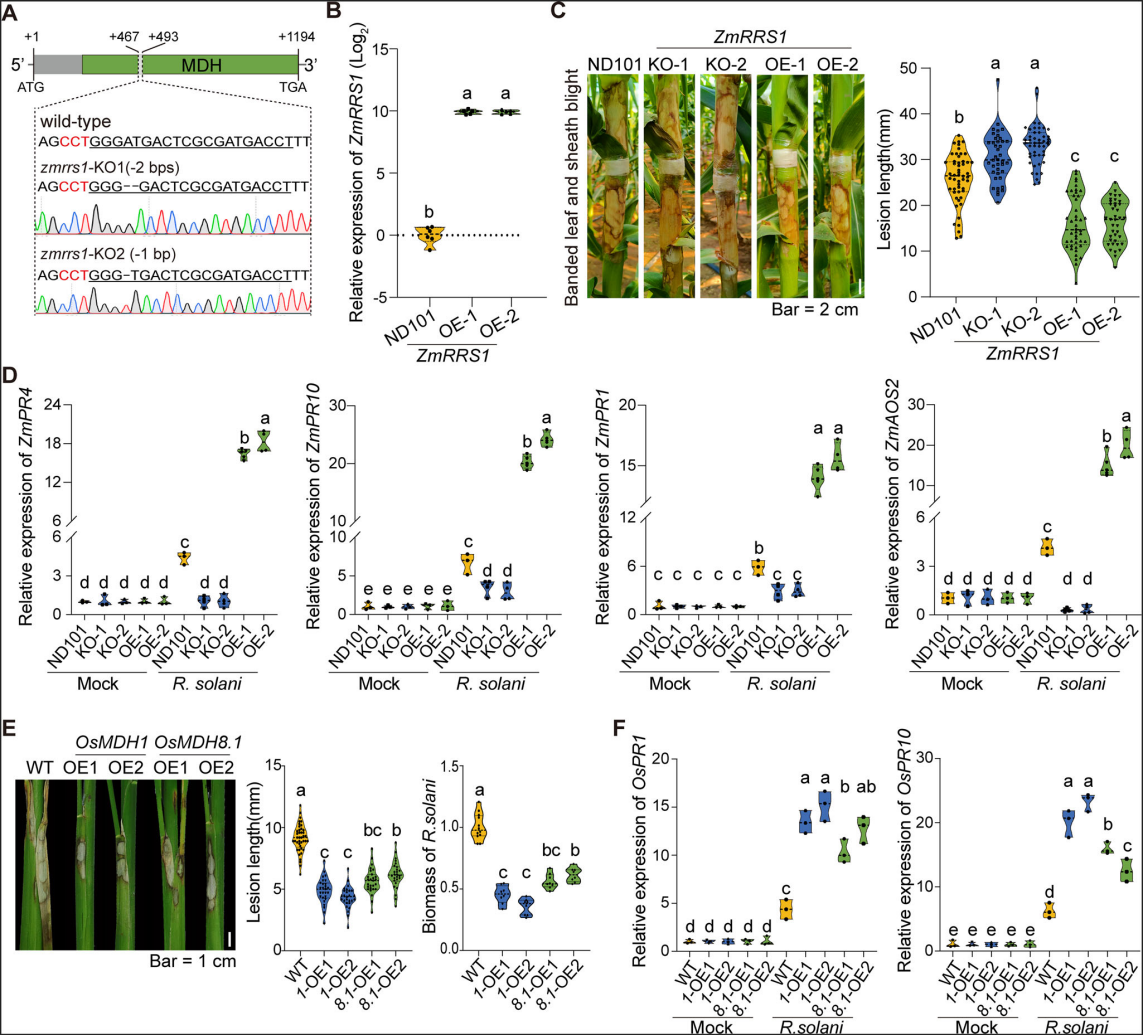
*ZmRRS1* and its homologs positively regulate *R. solani* resistance in maize and rice. A) *zmrrs1‐KOs* validation by Sanger sequencing. In the schematic, the exon is shown in gray, the malate dehydrogenase (MDH) domain in green. Under line and letter in red represent the gRNA target sequence and Protospacer Adjacent Motif (PAM), respectively. B) *ZmRRS1* transcript levels in WT and OE lines determined by RT‐qPCR (*n* = 5–6). C) Field phenotypes of leaf sheaths from wild‐type (WT), CRISPR/Cas9 knockouts (*zmrrs1‐*KO1 and KO2), and overexpression lines (*ZmRRS1*‐OE1 and OE2) at 10 dpi. Data represent *n* = 39–52 plants. Scale bars, 2 cm. D) Expression of defense‐related genes (*ZmPR4*, *ZmPR10*, *ZmPR1*, and *ZmAOS2*) in leaf sheaths of WT, KO, and OE plants at 24 hpi, as measured by RT‐qPCR (*n* = 3–5). E) Resistance phenotypes of rice plants (*Oryza sativa* cv. Nipponbare) overexpressing *OsMDH1* or *OsMDH8.1* at 7 dpi (left and middle). Leaf sheaths of two representative *OsMDH1*/*OsMDH8.1*‐OE lines are shown. Scale bars, 1 cm. *n* = 26–41. Right, relative biomass of *R. solani* in leaf sheaths quantified by RT‐qPCR using *R. solani*‐specific and *OsActin*‐specific primers (*n* = 8–12). F) Induction of rice defense genes (*OsPR1* and *OsPR10*) at 24 hpi in leaf sheaths of WT and OE lines, determined by RT‐qPCR (*n* = 3). In (B)–(F), data are presented as means ± s.e.m. Different lowercase letters indicate statistically significant differences (one‐way ANOVA followed by Tukey's test, *p* < 0.05). In the violin plots, the central line represents the median, the outline depicts the kernel density distribution, and overlaid dots represent individual data points. “Mock” indicates expression levels at 0 hpi.

To further confirm the role of *ZmRRS1* in maize resistance to *R. solani*, we generated two independent CRISPR/Cas9‐mediated mutant lines, *zmrrs1*‐KO1 and *zmrrs1*‐KO2 (ND101 background), which carry 2‐bp and 1‐bp deletions in the *ZmRRS1* coding region, respectively (**Figure** **2**A). Concurrently, we also developed two independent transgenic events overexpressing *ZmRRS1* in the ND101 background, both of which showed significantly elevated *ZmRRS1* expression levels (Figure 2B).

Following inoculation with *R. solani*, the knockout (KO) and overexpression (OE) lines, along with the ND101 line, were evaluated in the field, with lesion lengths measured at 10–14 days post‐inoculation (dpi). The two *zmrrs1‐*KO lines exhibited significantly longer lesions compared to ND101, while the two *ZmRRS1*‐OE lines displayed milder disease symptoms, reducing lesion length by ≈37.3% relative to ND101 (Figure 2C; Figure S2A, Supporting Information). Additionally, the expression levels of defense‐related genes *ZmPR4* (*GRMZM2G117942*), *ZmPR10* (*GRMZM2G112488*), *ZmPR1* (*GRMZM2G465226*), and a jasmonic acid biosynthesis enzyme encoding gene *ZmAOS2* (*GRMZM2G067225*) were significantly increased in *ZmRRS1*‐OE lines than in ND101 upon *R. solani* infection, while their expression was markedly reduced in the KO lines (Figure 2D). These findings suggest that *ZmRRS1* positively regulates resistance to BLSB in maize.

Given that *R. solani* also infects rice, we examined whether the rice homolog of *ZmRRS1* also confers *R. solani* resistance in rice. Phylogenetic analysis identified two rice homologs of *ZmRRS1*, *OsMDH1* and *OsMDH8.1*, both of which encode malate dehydrogenase (Figure S2B, Supporting Information). We generated transgenic rice lines overexpressing *OsMDH1* or *OsMDH8.1* in the Nipponbare background (Figure S2C,D, Supporting Information). At 7 dpi with *R. solani*, the *OsMDH1*‐ and *OsMDH8.1*‐overexpressing lines developed shorter lesions, reducing average lesion length by 4.5 and 3.2 cm, respectively, compared with Nipponbare (Figure 2E). Consistent with this, the expression of the rice defense genes *OsPR1* (*LOC_Os07g03730*) and *OsPR10* (*LOC_Os03g18850*) were highly upregulated in these overexpressing lines following *R. solani* infection (Figure 2F). Overall, these results suggest that *ZmRRS1* and its homologs function as conserved *R. solani* resistance genes in both maize and rice.

### 2.4 The 831‐bp Indel in the Promoter Determines the Differential *ZmRRS1* Expression and BLSB Resistance

**Figure 3:**
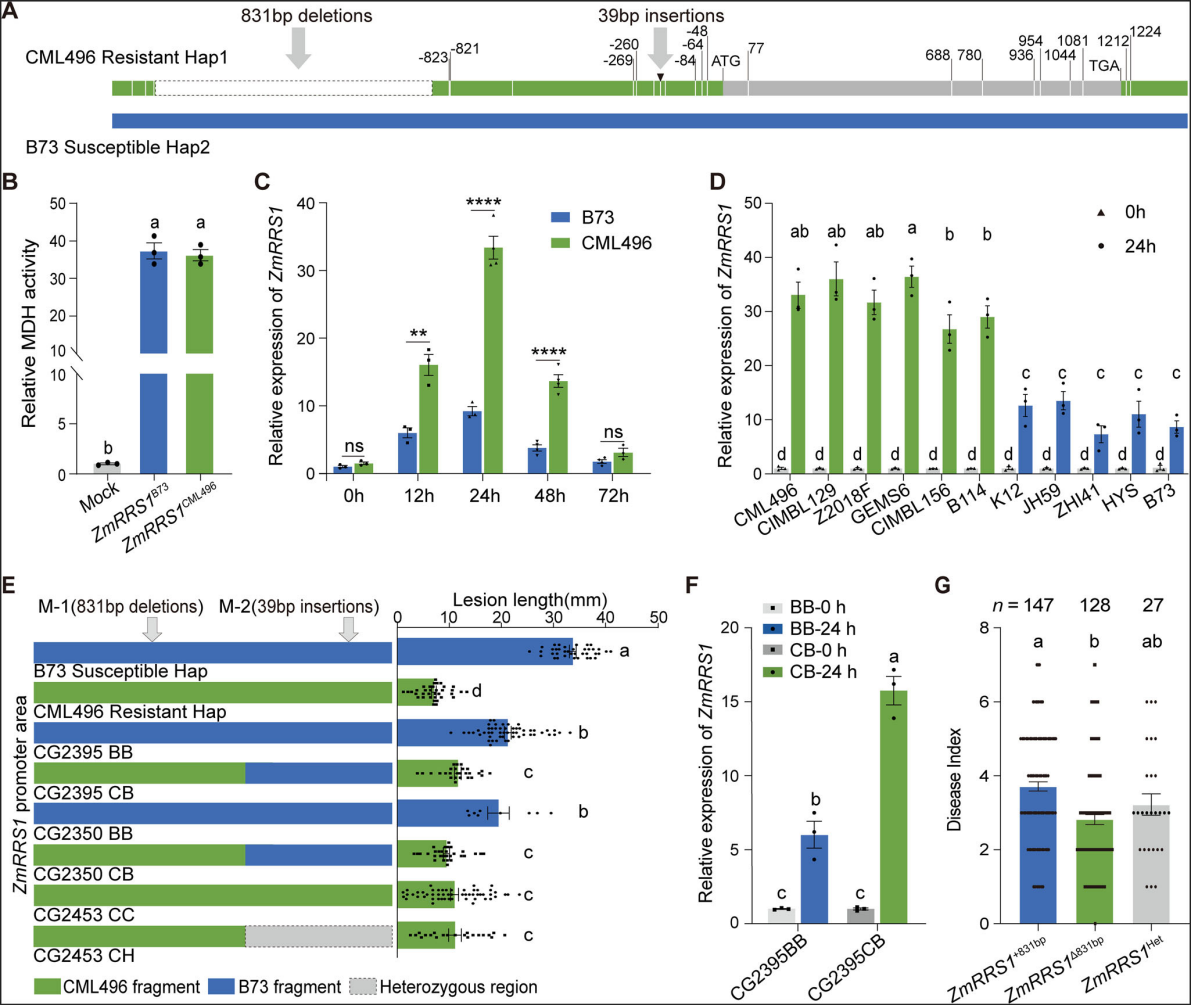
A natural 831‐bp indel regulates expression of *ZmRRS1* and BLSB resistance. A) Schematic representation of promoter and coding‐sequence polymorphisms distinguishing Hap1 (CML496) and Hap2 (B73). The 831‐bp deletion and 39‐bp insertion within the *ZmRRS1* promoter region are indicated by grey arrows. B) Malate dehydrogenase (MDH) enzymatic activity of recombinant ZmRRS1 proteins from CML496 and B73. C) Comparison of inducible *ZmRRS1* expression between the B73 (susceptible Hap2) and the CML496 (resistant Hap1). Gene expression levels were determined in leaf sheaths at 0, 12, 24, 48, and 72 h after *R. solani* infection by RT‐qPCR (*n* = 3–4). Data are presented as means ± s.e.m. Statistical significance was determined by two‐tailed Student's *t*‐test (ns, not significant; ** *p* < 0.01, **** *p* < 0.0001). D) Comparison of inducible *ZmRRS1* expression between the six resistant (Hap1, indicated in green) and five susceptible (Hap2, indicated in blue) maize inbred lines. Gene expression levels were determined in leaf sheaths at 24 hpi by RT‐qPCR (means ± s.e.m., *n* = 3; two‐way ANOVA with Tukey's test, *p* < 0.05). E) Association analysis in three BC_2_RIL‐derived HIF lines segregating at the *ZmRRS1* promoter. Left: HIF lines CG2395 and CG2350 (831‐bp deletion) and CG2453 (39‐bp insertion) were genotyped at the 831‐bp/39‐bp sites and grouped as BB (B73/B73), CC (CML496/CML496), CB (CML496 at 831 bp, B73 at 39 bp), or CH (CML496 at 831 bp, heterozygous at 39 bp). These classifications yielded three near‐isogenic line (NIL) pairs differing only at the *ZmRRS1* promoter. Right: Field sheath‐inoculation assays at 14 dpi revealed genotype‐dependent differences in lesion length (*n* = 9–53). F) Expression of *ZmRRS1* in leaf sheaths of NIL pairs (CG2395BB and CG2395CB) at 24 hpi, determined by RT‐qPCR (means ± s.e.m., *n* = 3; two‐way ANOVA with Tukey's test, *p* < 0.05). G) Association between BLSB disease index and *ZmRRS1* promoter genotypes (*ZmRRS1* ^Δ831bp^, *ZmRRS1* ^+831bp^, and *ZmRRS1* ^Het^) in a 302‐line maize diversity panel. A simple sequence length polymorphism (SSLP) marker targeting the 831‐bp insertion/deletion in the *ZmRRS1* promoter was used for genotyping and allele stratification. *n* denotes the allele frequency of *ZmRRS1* ^Δ831bp^ and *ZmRRS1* ^+831bp^ among the 302 maize accessions. In (B), (E), and (G), data are presented as means ± s.e.m. Different lowercase letters indicate statistically significant differences (one‐way ANOVA followed by Tukey's test, *p* < 0.05). RIL, recombinant inbred line; HIF, heterogeneous inbred family; NIL, near‐isogenic line.

To understand how *ZmRRS1* ^Hap1^ functions differently from *ZmRRS1* ^Hap2^ in BLSB resistance, we first analyzed the sequence variations of *ZmRRS1* within the maize NAM population. Compared to the B73 reference genome, this analysis revealed an 831‐bp deletion and a 39‐bp insertion in the promoter region of *ZmRRS1*, along with synonymous and nonsynonymous variations in the coding region (Figure S3A, Supporting Information). We then sequenced the genomic DNA of six resistant *ZmRRS1* ^Hap1^ (RH) lines and five susceptible *ZmRRS1* ^Hap2^ (SH) lines. Notably, the RH and SH lines had identical sequences to the corresponding resistance line CML496 and the susceptible line B73, respectively (**Figure** **3**A).

To determine if variations in the coding region affect protein function, we conducted malate dehydrogenase activity assays in vitro, which showed no significant differences between ZmRRS1^CML496^ and ZmRRS1^B73^ (Figure 3B). Additionally, both ZmRRS1^CML496^ and ZmRRS1^B73^ localize to the cytoplasm and chloroplasts (Figure S3B, Supporting Information). These results indicate that natural variations in the coding region do not impact the enzymatic activity or subcellular localization of ZmRRS1.

Given the substantial indels in the promoter region, we question whether these indels contribute to the functional differences between the two *ZmRRS1* haplotypes. There was no significant difference in the basal expression levels of *ZmRRS1* between the RH and SH groups (Figure S3C, Supporting Information). However, *ZmRRS1* was highly induced by *R. solani* treatment 24 hpi in the resistance line CML496, but weaker in the susceptible line B73 (Figure 3C). Consistently, the expression levels of defense‐related genes *ZmPR4* and *ZmPR10* were markedly elevated in CML496 relative to B73 upon *R. solani* infection (Figure S3D, Supporting Information). To validate this observation, we further randomly selected 11 inbred lines: 6 with the 831‐bp deletion and 39‐bp insertion in the promoter region from RH groups, and 5 lacking these variations from SH groups (Figure 3A). Compared to SH lines, the expression level of *ZmRRS1* was significantly higher in the RH lines following *R. solani* inoculation at 24 hpi (Figure 3D; Figure S3E, Supporting Information). Collectively, these findings suggest that variations in the promoter region leads to the differential *ZmRRS1* expression and BLSB resistance. Moreover, the *ZmRRS1* transcription levels are positively correlated with resistance to BLSB, which aligns with the above‐mentioned observation in *zmrrs1*‐KO and *ZmRRS1*‐OE lines (Figure 2A‐C).

To precisely identify the causal polymorphisms that explain the two functionally distinct *ZmRRS1* alleles, we performed a co‐segregation analysis using a BC_2_ recombinant inbred line (BC_2_RIL) population derived from the resistance line CML496 and the susceptible line Lx9801.^[^ ^47^ ^]^ Sanger sequencing revealed that the Lx980I shared identical *ZmRRS1* promoter and coding sequences with the representative Hap2 line B73. Among this population, we identified three HIF lines, including CG2395, CG2350, and CG2453, that are segregated for the promoter region of *ZmRRS1* (Figure 3E). This result showed that the 831 bp indel is significantly co‐segregated with the disease phenotype, whereas the 39 bp indel is not (Figure 3E). From the selfed progeny of HIF line CG2395, we developed two homozygous near‐isogenic lines (NILs), CG2395CB (NIL‐R; *ZmRRS1* ^Hap1^ allele across the 831‐bp) and CG2395BB (NIL‐S; *ZmRRS1* ^Hap2^ allele across the 831‐bp), and upon *R. solani* inoculation, observed significantly stronger *ZmRRS1* transcriptional induction in NIL‐R than in NIL‐S (Figure 3F). Consistently, the genome‐wide association reanalysis of 302 maize accessions further revealed that the 831‐bp indel exceeded the genome‐wide significance threshold and falls within the *ZmRRS1* LD block, supporting a strong association with resistance variation (Figure S3F, Supporting Information). The above results indicate that the 831 bp indel, instead of the 39 bp indel, contributes to the functional difference between the two *ZmRRS1* alleles.

Finally, we developed a simple sequence length polymorphism (SSLP) marker that distinguishes the 831‐bp indel between RH and SH lines (Figure S3G, Supporting Information). In the collection of 302 maize accessions, 128 (42.38 %) carry the *ZmRRS1* ^Δ831bp^ allele, 147 (48.68 %) possess the *ZmRRS1* ^+831bp^ allele and 27 (8.94 %) are heterozygous (Figure 3G). Association analysis revealed that *ZmRRS1* ^Δ831bp^ plants exhibited enhanced resistance to *R. solani* compared to *ZmRRS1* ^+831bp^ plants (Figure 3G), further supporting the association of the 831‐bp deletion with enhanced disease resistance.

### 2.5 The 831‐bp TE Element‐Induced Methylation Attenuates ZmWRKY44 Binding Affinity with *ZmRRS1* Promoter

**Figure 4:**
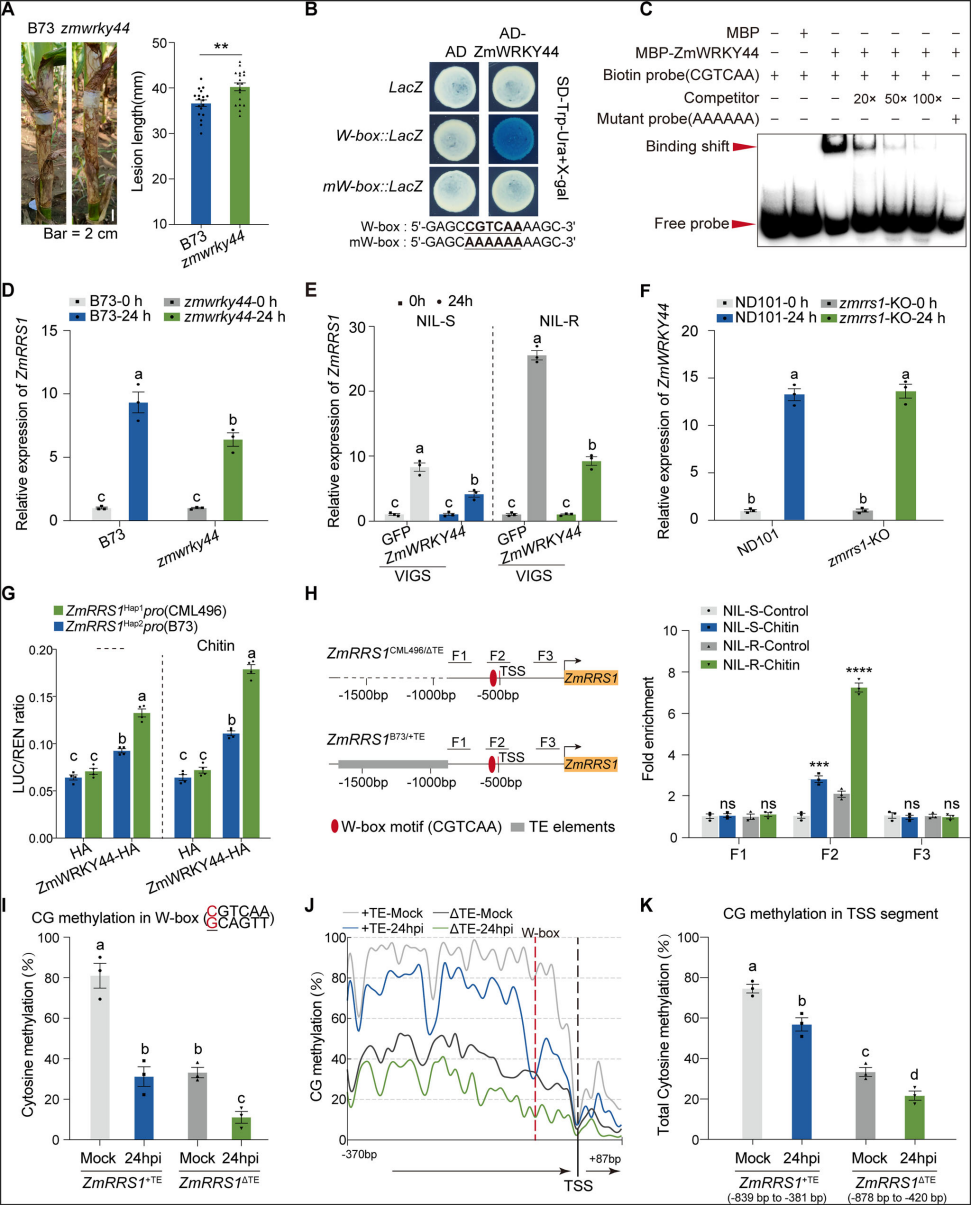
TE‐driven methylation interferes with ZmWRKY44 binding to the *ZmRRS1* promoter. A) Representative field phenotypes of leaf sheaths at 10 dpi in wild‐type B73 and the EMS‐derived mutant *zmwrky44* (*n* = 18). Scale bar, 2 cm. Data are presented as means ± s.e.m. Statistical significance was determined by two‐tailed Student's t‐test (**p < 0.01). B) Yeast one‐hybrid (Y1H) assay showing that ZmWRKY44 binds to the W‐box motif but not to a mutated W‐box within the *ZmRRS1* promoter region. C) Electrophoretic mobility shift assay (EMSA) demonstrating direct binding of ZmWRKY44 to the *ZmRRS1* promoter. Competition was assessed using unlabeled probes, with the mutated probe serving as control. D) *ZmRRS1* transcript levels in the EMS‐induced mutant *zmwrky44* at 0 and 24 hpi with *R. solani*, relative to wild‐type B73 (*n* = 3). E) *ZmWRKY44* was knocked down by VIGS in both NIL‐R and NIL‐S backgrounds, and *ZmRRS1* transcript levels were quantified at 0 and 24 hpi with *R. solani* by RT‐qPCR (*n* = 3). Plants infiltrated with the empty vector pCMV201‐2b_N81_‐GFP served as controls. F) *ZmWRKY44* transcript levels in homozygous *zmrrs1*‐KO lines at 0 and 24 hpi with *R. solani*, using ND101 wild type as the reference (*n* = 3). G) Transient dual‐luciferase (LUC) assay in maize protoplasts. Cells transfected with different construct combinations were incubated at 25 °C for 12 h, followed by chitin treatment for 10 min. Empty vector (EV‐HA) was used as a negative control (*n* = 4). H) Chromatin immunoprecipitation (ChIP)‐qPCR analysis of ZmWRKY44 binding to the *ZmRRS1* promoter in *ZmRRS1* ^Δ831bp^ and *ZmRRS1* ^+831bp^ haplotypes, with or without chitin. Left: Schematic of the 1.8‐kb *ZmRRS1* promoter. ATG (black arrow), TSS (red line) and W‐box (red oval) are indicated. The 831‐bp TE in the *ZmRRS1* ^+831bp^ promoter (NIL‐S) is shaded grey, whereas the homologous Δ831bp region in *ZmRRS1* ^Δ831bp^ (NIL‐R) is dashed. Black bars (F1‐F3) denote the qPCR amplicons. Right: Protoplasts expressing *ZmWRKY44*‐GFP or GFP were subjected to ChIP analysis using an anti‐GFP antibody. Enrichment of F1‐F3 regions was quantified by qPCR using site‐specific primers (*n* = 3). I) Mean CG methylation (%) at the W‐box motif within the *ZmRRS1* promoter in *ZmRRS1* ^ΔTE^ and *ZmRRS1* ^+TE^ haplotypes at 0 and 24 hpi (*n* = 3). J) Changes in CG methylation (%) across the 458‐bp TSS‐proximal region of the *ZmRRS1* promoter in *ZmRRS1* ^ΔTE^ and *ZmRRS1* ^+TE^ haplotypes at 0 and 24 hpi (*n* = 3). The x‐axis shows distance relative to the TSS, spanning 370 bp upstream and 87 bp downstream. The W‐box motif is marked by a red dashed line, and the TSS is indicated by a black dashed line. K) Mean CG methylation (%) across the 458‐bp region surrounding the TSS of the *ZmRRS1* promoter in *ZmRRS1* ^ΔTE^ and *ZmRRS1* ^+TE^ haplotypes at 0 and 24 hpi (*n* = 3). Distances plotted for the two *ZmRRS1* alleles beneath the x‐axis are given relative to the ATG start codon. In (D), (E), (F), (G), (H), (I) and (K), data are presented as means ± s.e.m. Different lowercase letters indicate statistically significant differences (two‐way ANOVA followed by Tukey's test, *p* < 0.05).

To search for the factors underlying the differential regulation of *ZmRRS1* expression, we resequenced the promoter fragment including the 831‐bp indel downstream of the transcription start site (TSS). In the promoter region, we identified several binding sites for various transcription factor families, including WRKY, LBD, ERF, and TALE (Table S4, Supporting Information). Notably, there is only one putative W‐box motif (5′‐CGTCAA‐3′) (Table S4, Supporting Information), a *cis* element recognized by WRKY transcription factors.^[^ ^48^ ^]^ Previous studies have reported a *WRKY* defense gene regulatory network (GRN) rapidly induced by *R. solani* in *Brachypodium distachyon*, among which *BdWRKY36* and *BdWRKY38* have been identified as two key positive resistance regulators shared among resistant varieties.^[^ ^49^ ^]^ In maize, two corresponding maize homologs, *ZmWRKY44* (*GRMZM2G432583*) and *ZmWRKY15* (*GRMZM2G004060*) were identified (Table S5, Supporting Information). qPCR analysis showed that *ZmWRKY44* was induced at 24 hpi by *R. solani* in both CML496 and B73 (Figure S4A, Supporting Information). By contrast, *ZmWRKY15* was up‐regulated at 24 hpi only in B73, whereas CML496 displayed no discernible induction (Figure S4B, Supporting Information). To this end, we selected ZmWRKY44 as a potential regulator of *ZmRRS1*. An ethyl methanesulfonate (EMS)‐induced mutant, *zmwrky44*, harboring a GT→AT splice‐donor mutation in intron 2 that drives intron retention and a premature stop within the WRKY DNA‐binding domain (Figure S4C,D, Supporting Information), displayed increased susceptibility after field inoculation with *R. solani* (**Figure** **4**A). In addition, silencing *ZmWRKY44* in both B73 and CML496 using the ZMBJ‐CMV‐VIGS tool also enhanced the disease susceptibility (Figure S4E,F, Supporting Information). These data indicate that ZmWRKY44 positively regulates maize BLSB resistance, consistent with previous observations.^[^ ^49^ ^]^

We next examined whether ZmWRKY44 binds to the putative W‐box in the *ZmRRS1* promoter to regulate its expression. Both *ZmRRS1* haplotypes encode an identical putative W‐box motif and adjacent sequences. The yeast one‐hybrid (Y1H) assay confirmed that ZmWRKY44 binds to this W‐box in the *ZmRRS1* promoter (Figure 4B). Recombinant ZmWRKY44 specifically binds to the *ZmRRS1* promoter fragment containing the putative W‐box, but not to the mutated W‐box. This binding was disrupted by competition with an unlabeled intact DNA probe, revealing that ZmWRKY44 directly binds to *ZmRRS1*, as shown by the electrophoretic mobility shift assay (EMSA) (Figure 4C). Therefore, we reasoned that ZmWRKY44 might directly regulate *ZmRRS1* expression. In the EMS‐derived *zmwrky44* line, *ZmRRS1* induction at 24 hpi was reduced relative to B73 (Figure 4D). Concordantly, VIGS‐mediated silencing of *ZmWRKY44* in both NIL‐S and NIL‐R attenuated the *R. solani*‐induced *ZmRRS1* transcription at 24 hpi and heightened disease susceptibility, with a stronger effect in NIL‐R (Figure 4E and Figure S5A,B, Supporting Information). In contrast, silencing *ZmWRKY44* in the *ZmRRS1*‐OE background did not erode resistance, which remained intact (Figure S5C,D, Supporting Information). *ZmWRKY44* transcript induction at 24 hpi was unchanged in the *zmrrs1*‐KO line (Figure 4F), placing *ZmWRKY44* upstream of *ZmRRS1* as a positive regulator. In transient transactivation assays, ZmWRKY44 activated both *ZmRRS1* ^Hap1^ and *ZmRRS1* ^Hap2^ promoters under mock and chitin treatment (Figure 4G and Figure S5E, Supporting Information). Together, these results demonstrate that ZmWRKY44 directly binds to the W‐box in the *ZmRRS1* promoter to positively modulate its expression.

The above observations prompted us to further investigate whether ZmWRKY44 differentially binds the promoter of the two *ZmRRS1* haplotypes to regulate its expression for BLSB resistance. To test this hypothesis, we conducted a chromatin immunoprecipitation followed by quantitative PCR (ChIP‐qPCR) by transiently expressing the ZmWRKY44‐GFP construct in maize protoplasts derived from inbred lines carrying either *ZmRRS1* ^Hap1^ (NIL‐R) or *ZmRRS1* ^Hap2^ (NIL‐S). The results showed that ZmWRKY44 was enriched at the W‐box, confirming its direct binding to the promoter of *ZmRRS1* in *vivo* (Figure 4H; Figure S5F, Supporting Information). Notably, we observed a stronger binding affinity of ZmWRKY44 to the W‐box motif in *ZmRRS1* ^CML496^ promoter (with the 831‐bp indel) compared with the *ZmRRS1* ^B73^ promoter (Figure 4H). This differential binding affinity was further enhanced by treatment of chitin, a known fungal PAMP that elicits plant basal defense responses (Figure 4H). Collectively, these results suggest that the 831‐bp indel in the *ZmRRS1* promoter affects the binding affinity of WRKY transcription factors such as ZmWRKY44, which is further enhanced in response to fungal PAMP treatment.

MaizeGDB annotates the 831‐bp fragment as a Tc1/Mariner superfamily, a kind of type II “cut‐and‐paste” transposable element (TE) that is widely distributed in nature.^[^ ^50^ ^]^ Such TEs are typically silenced by dense DNA methylation reinforced by repressive histone marks. Mounting evidence indicates that promoter‐inserted TEs can extend their methylation into adjacent regions, blocking transcription‐factor binding sites and reducing gene expression.^[^ ^36^, ^51^ ^]^ To test whether this mechanism constrains *ZmRRS1*, we quantified the methylation levels of two NIL lines, NIL‐S (*ZmRRS1* ^+TE^) and NIL‐R (*ZmRRS1* ^ΔTE^). Targeted bisulfite sequencing revealed that, under mock conditions, the ZmWRKY44 W‐box (CGTCAA) was hyper‐methylated in *ZmRRS1* ^+TE^ relative to *ZmRRS1* ^ΔTE^ (Figure 4I). At 24 hpi with *R. solani*, methylation at this site declined in both genotypes, with a more pronounced reduction in *ZmRRS1* ^ΔTE^ (Figure 4I). Genome‐wide cytosine profiling around the TSS revealed a similar trend (Figure 4J,K; Figure S5G, Supporting Information), reflecting changes in ZmWRKY44 ChIP‐qPCR enrichment (Figure 4H). In addition, pathogen challenge also attenuates methylation of the promoter in both *ZmRRS1* alleles (Figure 4J,K). Overall, these results suggest that TE‐dependent promoter methylation inhibits ZmWRKY44 binding to the W‐box motif, thereby reducing the expression of the *ZmRRS1* gene.

### 2.6 *ZmRRS1* Modulates the Pathways Related to ROS Homeostasis

**Figure 5:**
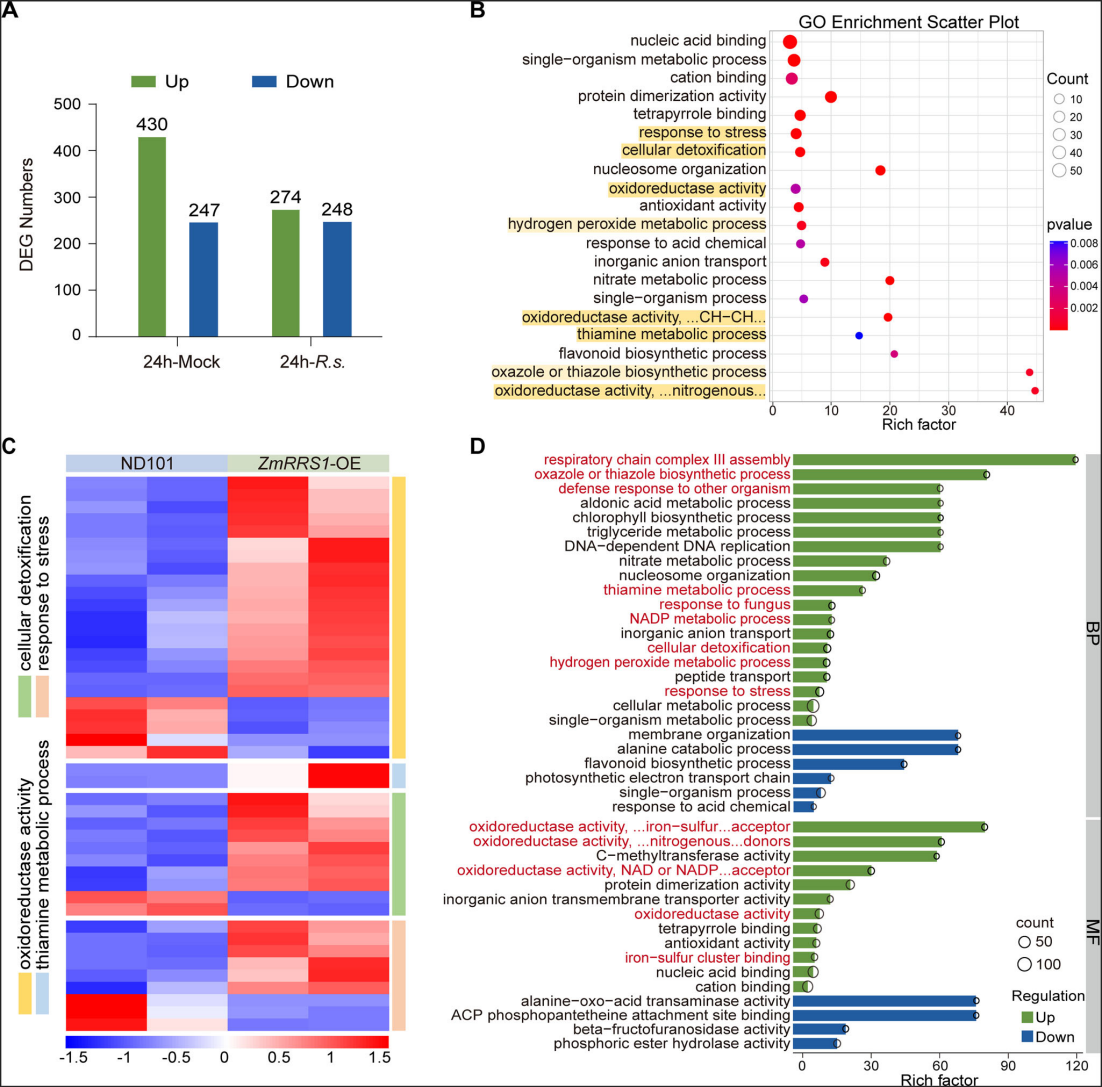
*ZmRRS1* over‐expression enhances ROS‐mediated defense pathways. A) Transcriptome analysis of differentially expressed genes (DEGs) regulated by *ZmRRS1*. Leaf sheaths of wild‐type ND101 and *ZmRRS1*‐OE plants were collected at 24 hpi with *R. solani* or water (mock). DEGs were defined by *p* < 0.05 and |log_2_(fold change)| ≥ 1.5; green bars indicate up‐regulated and blue bars down‐regulated genes. B) Gene Ontology (GO) enrichment of *ZmRRS1*‐dependent DEGs. The bubble plot shows the top 20 GO terms ranked by adjusted *P*‐value (*p*_adj_). The x‐axis indicates the enrichment ratio, and the y‐axis lists GO terms. Bubble size corresponds to the number of DEGs annotated per term; color reflects *p*_adj_ values. GO terms most relevant to this study are highlighted in orange. C) Cluster heatmap of DEGs involved in plant disease resistance‐related pathways. Red and blue indicate high and low expression levels, respectively. D) GO enrichment analysis of up‐ and down‐regulated *ZmRRS1*‐dependent DEGs. The bar chart ranks the top 41 GO terms by *p*_adj_. Bars are color‐coded by direction of regulation (green, up‐regulated; blue, down‐regulated). BP, biological process; MF, molecular function. Data in (B)–(D) were obtained at 24 hpi with *R. solani*.

To elucidate the signaling pathways underlying *ZmRRS1*‐mediated resistance to *R. solani*, we performed transcriptome sequencing (RNA‐seq) on ND101 (wild type, WT) and *ZmRRS1* overexpressing (OE) plants, inoculated or mock‐inoculated for 24 h (abbreviated as 24h‐*R.s*. and 24h‐Mock, respectively). Comparative analyses of differentially expressed genes (DEGs) between ND101 and *ZmRRS1*‐OE lines identified 1199 *ZmRRS1*‐associated DEGs (677 at 24h‐Mock and 522 at 24h‐*R.s*.), 101 of which were shared across contrasts (FDR < 0.05 and |log_2_(fold change)| ≥ 1.5; **Figure** **5**A; Tables S6 and S7, Supporting Information). Among the 522 DEGs at 24h‐*R.s*., 274 were upregulated and 248 were downregulated in *ZmRRS1*‐OE plants relative to ND101 (Figure 5A; Figure S6A, Supporting Information). Gene Ontology (GO) enrichment analysis revealed that most DEGs are mapped to defense‐related pathways, particularly those associated with reactive oxygen species (ROS) homeostasis. Key GO terms included response to stress (GO:0 006950), cellular detoxification (GO:1 990 748), oxidoreductase activity (GO:00 16491), hydrogen peroxide metabolic process (GO:00 42743), thiamine metabolic process (GO:0 006772), and the oxazole or thiazole biosynthetic process (GO:00 18131) (Figure 5B,C). Notably, most DEGs related to biological processes (BP) and molecular functions (MF) were upregulated (Figure 5D), indicating increased oxidoreductase activity and enhanced defense responses. Overall, *ZmRRS1* overexpression confers improved resistance to *R. solani* primarily by activating defense pathways and reprogramming redox metabolism to regulate ROS homeostasis.

Under non‐inoculated conditions, we identified 677 DEGs between *ZmRRS1*‐OE and ND101, including 430 upregulated and 247 downregulated genes (Figure 5A). GO and Kyoto Encyclopedia of Genes and Genomes (KEGG) enrichment analyses of these mock‐treated samples revealed that *ZmRRS1*‐OE plants showed enhanced pathways related to plant defense and stress responses, including oxidoreductase activity and response to stress pathways (GO), as well as plant hormone signal transduction (ko04075), MAPK signaling pathway‐plant (ko04016), phenylpropanoid biosynthesis (ko00940) and amino sugar and nucleotide sugar metabolism (ko00520) (KEGG) (Figure S6B–E, Supporting Information). Hierarchical clustering of these DEGs cleanly separated ND101 from *ZmRRS1*‐OE under mock conditions (Figure 5A; Figure S6F, Supporting Information).

### 2.7 *ZmRRS1* Enhances Mitochondria‐Derived ROS Accumulation

**Figure 6:**
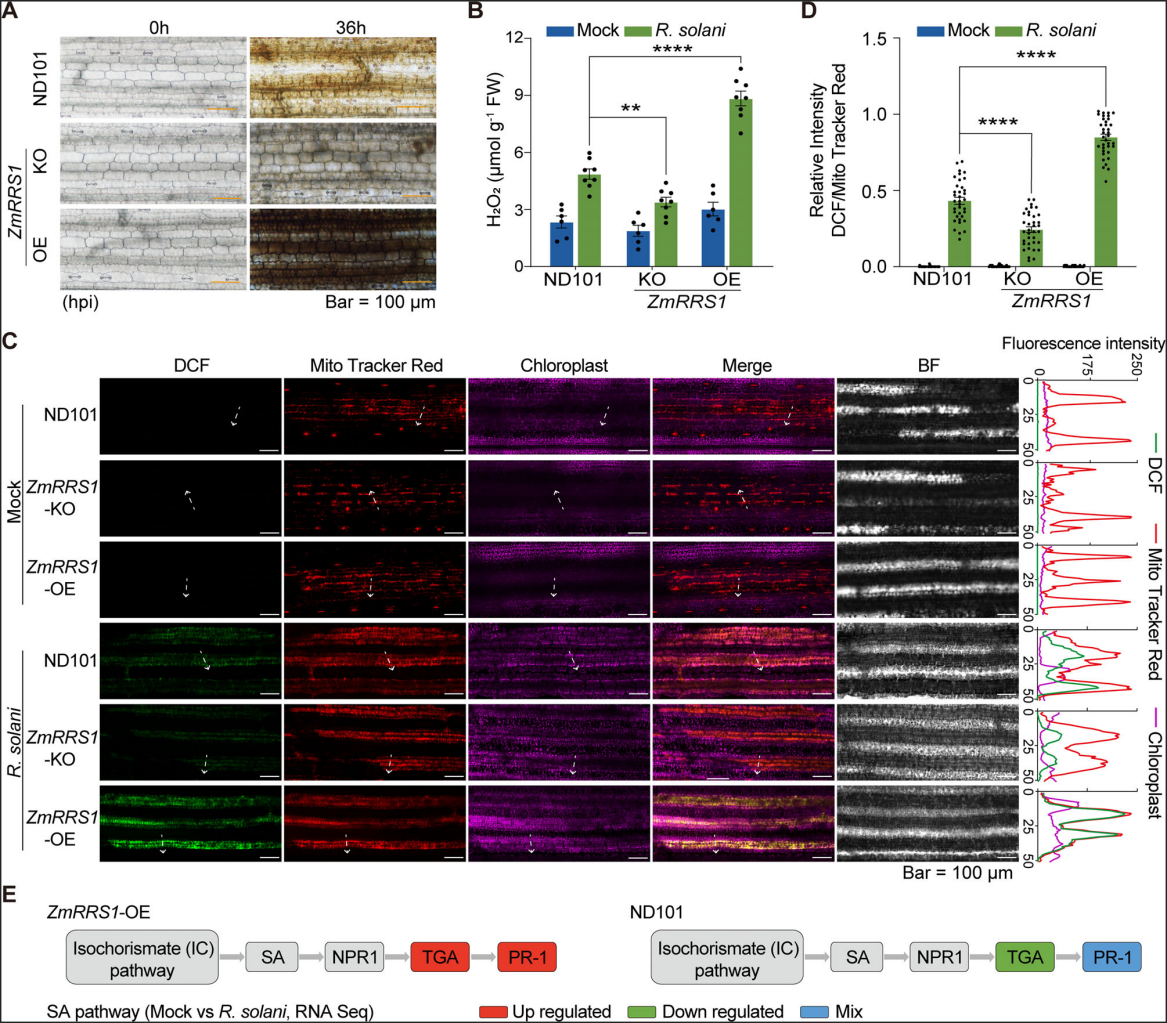
*ZmRRS1* promotes the accumulation of mitochondria‐derived ROS and engages salicylic acid signaling during *R. solani* infection. A) 3,3′‐Diaminobenzidine (DAB) staining of leaves from different transgenic lines after 36 h of *R. solani* infection. H_2_O_2_ accumulation is visualized as dark‐brown deposits. Scale bar, 100 µm. B) Quantification of H_2_O_2_ content in WT, *zmrrs1*‐KO, and *ZmRRS1*‐OE plants 36 h after *R. solani* inoculation. Water‐treated plants served as mock controls (*n* = 6–8). C) Subcellular localization of ROS in ND101, *zmrrs1*‐KO, and *ZmRRS1*‐OE leaves under *R. solani* infection and mock treatment. Signals for DCF‐labeled H_2_O_2_ (green), MitoTracker Red–stained mitochondria (red), and chlorophyll autofluorescence (magenta) were recorded at 36 hpi with *R. solani* and in mock controls to assess signal overlap. White dashed arrows mark the line‐scan paths; the corresponding fluorescence intensity profiles are shown to the right (green, DCF–H_2_O_2_; red, MitoTracker Red; magenta, chlorophyll). Scale bar, 100 µm. D) Quantification of DCF–H_2_O_2_ fluorescence in panel C, normalized to the MitoTracker Red mitochondrial signal (*n* = 36). (E) Salicylic acid (SA) KEGG pathway analysis of RNA‐seq data at 24 hpi with *R. solani* in *ZmRRS1*‐OE and the ND101 background. Compared with ND101, *ZmRRS1*‐OE shows differential enrichment in the SA responsive pathway at 24 hpi. DEGs for KEGG analysis were defined as *p* < 0.05 and |log_2_(fold change)| ≥ 1.5. Upregulated pathways are marked in red, downregulated in green, and pathways containing both up‐ and downregulated genes in blue. In (B) and (D), data are presented as means ± s.e.m. (one‐way ANOVA followed by Tukey's test, ** *p* < 0.01, **** *p* < 0.0001).

Given the enrichment of ROS homeostasis‐related genes in response to *R. solani*, we measured H_2_O_2_ levels in *ZmRRS1‐*KO and OE lines during infection. DAB staining revealed elevated H_2_O_2_ in *ZmRRS1*‐OE and reduced levels in *zmrrs1*‐KO relative to ND101 (**Figure** **6**A **;** Figure S7A, Supporting Information). Direct quantification of H_2_O_2_ further corroborated these results (Figure 6B). Similar trends were observed in resistant CML496 (carrying *ZmRRS1* ^Hap1^) and susceptible B73 (carrying *ZmRRS1* ^Hap2^). At 36 hpi, H_2_O_2_​ measurements and DAB staining likewise showed enhanced ROS accumulation in CML496 relative to B73, mirroring patterns seen in *ZmRRS1*‐KO and OE lines (Figure S7B,C, Supporting Information). Collectively, these results indicate that *ZmRRS1* confers resistance to *R. solani* by promoting ROS accumulation in maize.

In *Arabidopsis*, excess chloroplast NADH is oxidized by plastidic NAD‐MDH to malate, which is exported via the dicarboxylate transporter *DiT1* and re‐oxidized by mitochondrial MDH to generate matrix NADH that drives Complex I‐dependent mitochondrial ROS (mtROS); import of NAD⁺ via the mitochondrial carrier *NDT2* gates this flux.^[^ ^52^, ^53^ ^]^ This “malate shuttle/circulation” provides a conserved plastid‐to‐mitochondrion redox relay linking chloroplast metabolism to mtROS output.^[^ ^31^ ^]^ Given that *ZmRRS1* encodes a chloroplast/cytosol NAD‐MDH, we hypothesized that pathogen‐triggered *ZmRRS1* overexpression would potentiate plastid‐derived reductant flow into mitochondria and elevate mtROS through this pathway. To test this, we first profiled maize homologs of *DiT1*, *mMDH1*, and *NDT2*: all three were significantly induced at 24 hpi with *R. solani* in *ZmRRS1*‐OE lines relative to mock, consistent with activation of the malate valve (Figure S7D, Supporting Information).^[^ ^31^ ^]^ To precisely capture the subcellular localization of pathogen‐induced ROS, maize leaves with different genotypes were double‐stained with the mitochondria‐specific marker MitoTracker Red CMXRos and the ROS probe H_2_DCFDA, a non‐fluorescent compound that readily enter cells and form H_2_DCF in the presence of endogenous esterases and H_2_O_2_. The results showed that pathogen‐induced ROS, namely H_2_O_2_ measured with CM‐H_2_DCFDA (DCF), broadly overlapped with MitoTracker Red–labeled mitochondria but not with chlorophyll autofluorescence (Figure 6C). Quantitative measurement showed that ROS production is strongly induced in *ZmRRS1*‐OE lines, slightly in ND101 wild type plants and barely in *zmrrs1* mutants (Figure 6D). This is consistent to DAB staining and H_2_O_2_ quantification in Figure 6A,B. Together, these data support a model in which *R. solani* infection enhances *ZmRRS1*‐driven plastid‐to‐mitochondrion electron transfer via the malate shuttle, resulting in Complex I‐linked mtROS and downstream defense activation, extending the plastid‐mitochondrion redox relay paradigm to maize immunity.

In parallel, local H_2_O_2_ has been shown to sulfenylate the transcription factor CCA1 HIKING EXPEDITION (CHE), thereby activating the isochorismate (IC) branch at ISOCHORISMATE SYNTHASE1 (ICS1) and elevating salicylic acid (SA) in neighboring/systemic tissues.^[^ ^54^ ^]^ Consistent with this H_2_O_2_‐SA module, RNA‐seq at 24 hpi revealed significant upregulation of SA‐response nodes TGA (TGACG motif‐binding factor, a basic leucine zipper transcription factor) and PR‐1 (Pathogenesis‐Related protein 1) in *ZmRRS1*‐OE plants, whereas ND101 showed a predominantly downward trend (Figure 6E). Targeted qPCR further corroborated stronger induction in OE relative to ND101/KO for the IC‐pathway gene *ZmICS1* and for SA‐signaling outputs *ZmNPR1* (Nonexpressor of Pathogenesis‐Related genes 1), *ZmPR1* (Pathogenesis‐Related protein 1)/*ZmPR2* (Pathogenesis‐Related protein 2; β‐1,3‐glucanase), and *ZmbZIP39* (Octopine synthase element–binding factor 1, OBF1, a TGA‐class bZIP transcription factor), in agreement with the RNA‐seq data (Figure 2D; Figure S7E–H, Supporting Information).

### 2.8 The *ZmRRS1*‐KO and OE Lines Exhibit No Agronomic Penalty

**Figure 7:**
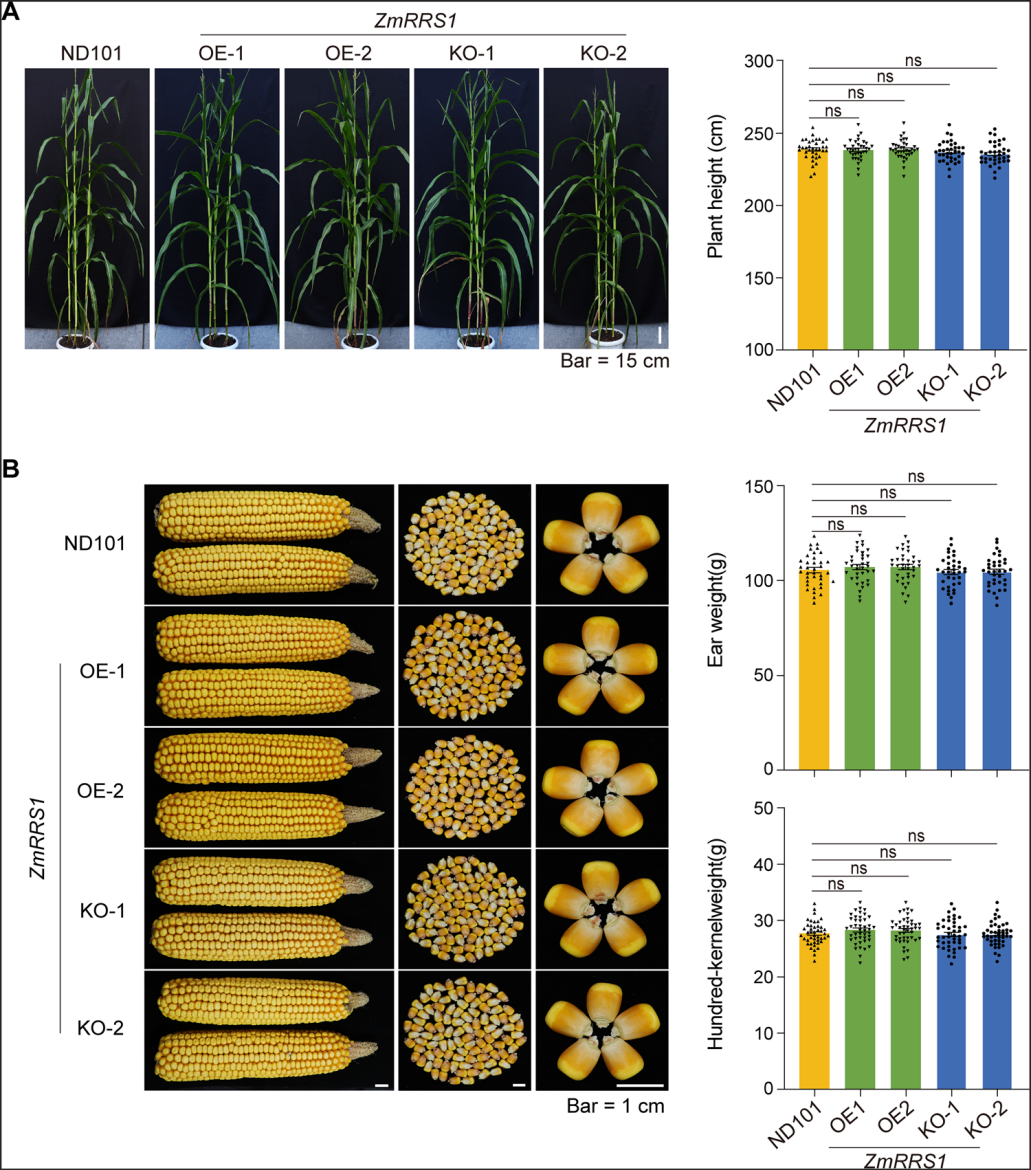
Knock‐out and overexpression of *ZmRRS1* have no negative effects on the key agronomic traits of maize. A) Field performance of *ZmRRS1* transgenic lines at maturity under normal growth conditions. Left: representative whole‐plant morphology of wild type (WT), *ZmRRS1* knockout (KO), and overexpression (OE) lines grown in the field. Right: quantification of plant height (*n* = 36). Scale bar, 15 cm. B) Yield‐related traits of *ZmRRS1* transgenic lines under normal conditions. Left: representative ear, 100‐kernel, and single‐kernel morphology of *zmrrs1*‐KO and *ZmRRS1*‐OE lines compared with WT; no visible differences observed. Scale bar, 1 cm. Right: bar charts showing ear weight and 100‐kernel weight of WT, KO, and OE lines (*n* = 36–44). In (A) and (B), data are presented as means ± s.e.m. Statistical significance was determined by one‐way ANOVA followed by Tukey's test (*p* < 0.05; ns, not significant).

To assess the impact of *ZmRRS1* on key agronomic traits, we evaluated the growth of *ZmRRS1*‐OE and KO lines alongside wild‐type controls under field conditions. The analysis indicated that both OE and KO lines displayed growth characteristics comparable to those of wild‐type plants, including plant height and ear height (**Figure** **7**A **;** Figure S8A, Supporting Information). Additionally, no significant differences were observed in key agronomic traits, such as ear weight, hundred‐kernel weight, ear dimensions, and grain size, among the OE, KO lines, and wild type (Figure 7B; Figure S8A,B, Supporting Information). These results suggest that *ZmRRS1* enhances resistance to BLSB without affecting growth and yield under normal conditions.

## 3 Discussion

**Figure 8:**
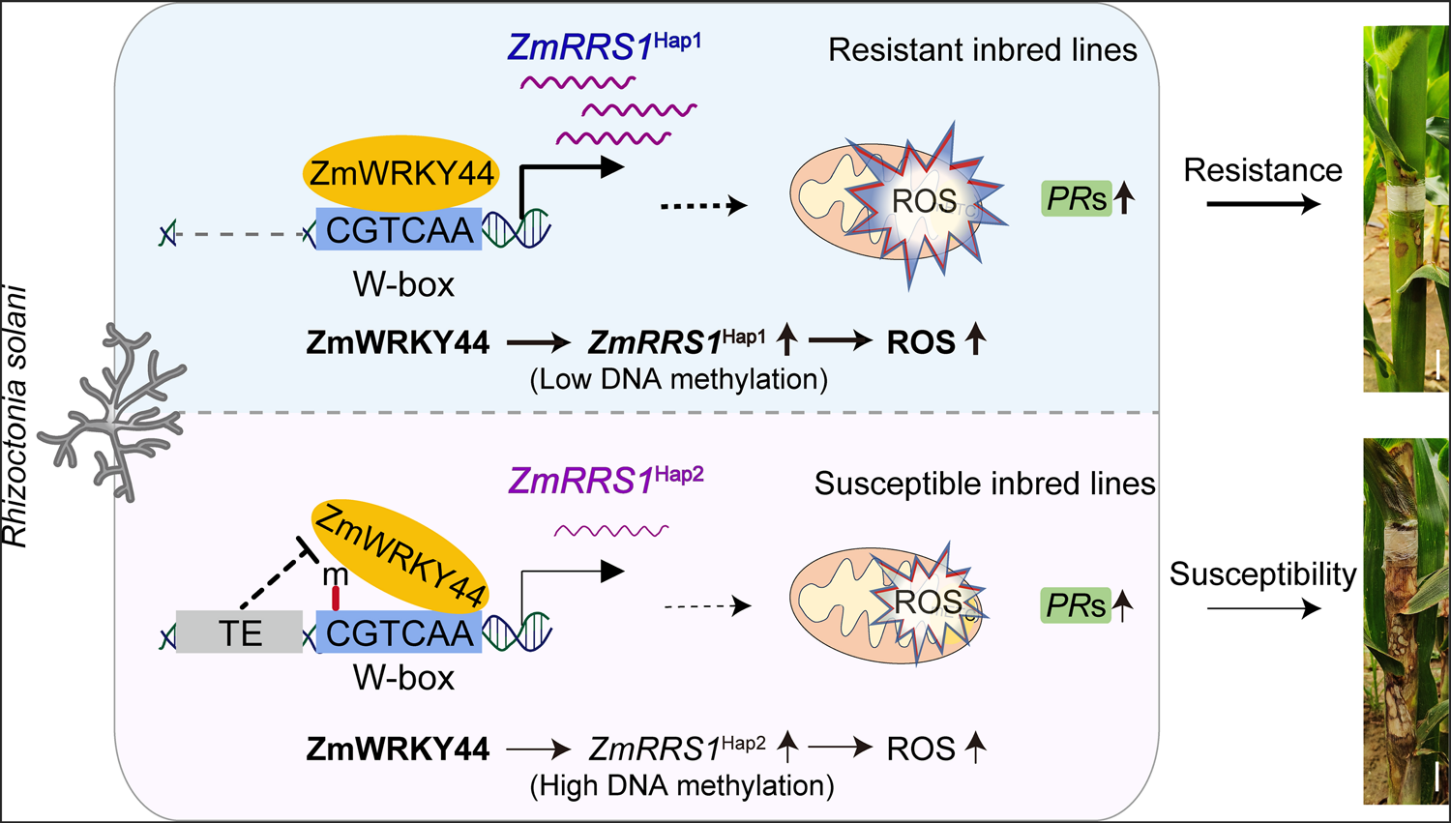
Working model of *ZmRRS1*‐mediated maize resistance to BLSB. In resistant Hap1 lines lacking the TE insertion, low promoter methylation permits stable ZmWRKY44 binding, thereby enabling rapid pathogen‐induced *ZmRRS1* activation, a stronger mitochondria‐derived ROS burst, and robust induction of downstream pathogenesis‐related genes. By contrast, in susceptible Hap2 inbred lines, insertion of an 831‐bp transposable element into the *ZmRRS1* promoter drives dense DNA methylation, which blocks ZmWRKY44 binding to the adjacent W‐box motif and markedly attenuates *R. solani*‐induced *ZmRRS1* transcription. The heightened ROS‐mediated defense restricts fungal progression without penalizing key agronomic traits. Schematic of differential regulation of the ZmWRKY44‐ZmRRS1‐mitochondrial ROS module between resistant and susceptible haplotypes is shown at the bottom of each panel, respectively.

Banded leaf and sheath blight (BLSB), caused by *R. solani*, is a pervasive and highly destructive disease that jeopardizes maize production worldwide. In this study, we identify the *ZmRRS1* gene, which encodes a maize malate dehydrogenase, as a significant quantitative locus that governs BLSB resistance in maize. *ZmRRS1* enhances the early ROS burst triggered by *R. solani* invasion, thereby bolstering BLSB resistance. Natural variation in the superior *ZmRRS1* ^Hap1^ allele, loss of an 831‐bp TE, diminishes TE‐mediated promoter methylation, allowing stronger recruitment of WRKY factors such as ZmWRKY44 and, in turn, elevating *ZmRRS1* transcription to fortify BLSB resistance (**Figure** **8**). Conceptually, this pathway is distilled to a regulatory module ZmWRKY44‐ZmRRS1‐ROS that links WRKY‐driven transcriptional activation to a defense‐amplifying redox pulse. Notably, the *ZmRRS1* ^Hap1^ allele provides this enhanced immunity without any detectable drawbacks to plant growth or yield.

### 3.1 *ZmRRS1* Extends MDH Function from Redox Metabolism to Plant Disease Resistance

Our data identify *ZmRRS1*, a malate dehydrogenase (MDH), as a key determinant of maize resistance to *R. solani*, positioning MDH as a direct mediator of biotic stress adaptation. Unlike most prior work emphasizing abiotic stress,^[^ ^25^, ^26^, ^29^ ^]^ recent studies link MDH isoforms to immunity: plastidial NAD‐MDH‐driven malate export seeds mitochondria‐derived ROS and PCD, whereas cytosolic MDH potentiates salicylic‐acid (SA)‐dependent defense.^[^ ^30^, ^31^, ^52^, ^53^ ^]^ Consistent with this, the dual cytosolic/chloroplastic ZmRRS1 functions as a redox hub coupling primary metabolism to defense signaling during infection. *ZmRRS1* overexpression elicits an early ROS burst and canonical PR‐gene activation, enhancing resistance and establishing *ZmRRS1* as a positive immune regulator. The conservation of its rice orthologue (*OsMDH1/8.1*) underscores the portability of MDH‐centered redox‐immunity modules across cereals, and the WRKY‐MDH‐ROS circuit described here provides a blueprint for mining quantitative resistance loci in other crops.

The *ZmRRS1* emerges as a novel regulator of maize immunity against *R. solani*. Within hours of infection, the resistant *ZmRRS1* ^Hap1^ allele triggers a pronounced oxidative burst that limits early pathogen ingress, reinforcing the front‐line role of ROS in plant immunity.^[^ ^17^ ^]^ Upstream perception by receptor‐kinase complexes (ZmWAKL‐ZmWIK‐ZmBLK1‐ZmRBOH4; ZmLecRK1‐ZmBAK1) initiates apoplastic ROS,^[^ ^23^, ^55^ ^]^ midstream modulators tune amplitude (the F‐box ZmFBL41 targeting ZmCAD and ZmNCED6; ZmPUB19 relieving the MAPK5 brake),^[^ ^9^, ^11^ ^]^ and downstream amplifiers such as *ZmRRS1* boost oxidative and transcriptional outputs without growth penalties. Light‐induced phosphorylation of LHCB5 in rice similarly intensifies chloroplast ROS and broad‐spectrum blast resistance.^[^ ^56^ ^]^ These layered controls converge to regulate immunity at distinct subcellular sites. ROS‐SA crosstalk further integrates the cascade: local H_2_O_2_ sulfenylates CHE, engaging the isochorismate branch at ICS1 and elevating SA locally and systemically, while SA‐activated NPR1 restrains excessive PCD to promote survival.^[^ ^54^, ^57^ ^]^ In line with this H_2_O_2_‐SA‐NPR1 module, our 24‐hpi RNA‐seq and targeted qPCR show induction of SA biosynthesis/signaling markers in *ZmRRS1*‐OE plants following *R. solani* challenge, indicating that SA‐pathway activation contributes to BLSB resistance in the *ZmRRS1*‐OE background.

### 3.2 TE‐Mediated Epigenetic Silencing of *ZmRRS1* Regulates Maize Immunity

An 831‐bp TE in the *ZmRRS1* promoter functions as a natural epigenetic silencer, dampening *ZmRRS1* transcription and reshaping BLSB resistance. In plants, DNA methylation and repressive histone marks (e.g., H3K9me2) cooperate to silence transposons and safeguard genome integrity,^[^ ^33^ ^]^ and this repression can spread into nearby regulatory DNA when a TE inserts close to a promoter.^[^ ^58^ ^]^ Through transcriptional or post‐transcriptional silencing, TEs recruit complexes that deposit dense DNA methylation and H3K9me2,^[^ ^51^ ^]^ forming heterochromatin that invades adjacent promoters, masks TF motifs, and blocks activator or basal‐machinery recruitment.^[^ ^36^, ^59^ ^]^ Thus, a promoter‐proximal TE can repress neighboring genes at both genetic and epigenetic layers with phenotypic consequences.^[^ ^38^, ^40^, ^60^ ^]^

Mechanistically, symmetric CpG methylation is sufficient to impede WRKY access at the *ZmRRS1* W‐box. 5mC at the first cytosine of the forward strand “CGTCAA” implies methylation of the complementary cytosine (“GCAGTT”), mapping to the W‐box core TGAC (C4). Single‐site 5mC at C4 creates a steric clash between the 5‐methyl group in the major groove and the conserved tyrosine in the WRKY β2 strand, repelling WRKY binding and blunting *ZmRRS1* induction.^[^ ^61^ ^]^ Consistently, a maize pan‐cistrome analysis showed that 98.2% of TF binding sites exhibit higher occupancy at the hypomethylated allele, indicating a pervasive negative association between mCG/mCHG and TF binding.^[^ ^62^ ^]^ Genome‐wide methylation profiling around the TSS (Figure 4J) further suggests that TE‐directed methylation modulates additional TFs at the *ZmRRS1* promoter, contributing to the distinct transcriptional responses of resistant and susceptible haplotypes during *R. solani* infection. In *ZmRRS1*, TE‐associated silencing likely reduces expression and weakens resistance, reminiscent of *ZmCCT*, where a promoter TE elevates GC methylation, dampens inducible transcription, and increases susceptibility to *Gibberella* stalk rot.^[^ ^63^ ^]^ Whether auxiliary layers (e.g., H3K9me2 spreading or reduced chromatin accessibility) amplify this repression remains to be determined. Notably, plants appear to exploit TE‐induced epigenetic variation as adaptive trait diversity.^[^ ^64^ ^]^

TE insertions broadly tune disease resistance across crops. In rice, an upstream LTR retrotransposon (“Renovator”) donates promoter activity to the ancient NLR *Pit*, restoring race‐specific blast resistance.^[^ ^65^ ^]^ At *Pigm*, tandem MITEs recruit RdDM‐linked methylation to enforce pollen‐biased repression of *PigmS*, quantitatively tempering the antagonistic NLR pair and balancing yield.^[^ ^66^ ^]^ In sorghum, promoter‐proximal MITEs combined with loss of the antisense NAT (*CARG*) elevate *ARG1* and confer broad‐spectrum fungal resistance.^[^ ^67^ ^]^ In cucumber, a non‐autonomous LTR in the susceptibility gene *CsaMLO8* disrupts splicing, creating a natural recessive allele for powdery‐mildew resistance.^[^ ^68^ ^]^ At macro‐evolutionary scale, LTR‐mediated retroduplication expanded pepper NLR repertoires, diversifying immune receptors.^[^ ^69^ ^]^ Collectively, TE polymorphisms can modulate immune regulators, remodel chromatin, or inactivate S genes, thereby generating selectable expression and functional diversity. Under fluctuating pathogen pressure, selection toggles TE⁺/TE^−^ and loop‐forming haplotypes to drive adaptive immune diversification; practically, TE‐linked *cis* variants, removing a silencing TE (e.g., *ZmRRS1*), or harnessing TE‐borne modules and chromatin logic (e.g., *Pit*/*Pigm*; *RPP7*/*RPP4*/*EFR*), are actionable targets for breeding durable resistance with minimal fitness costs.^[^ ^39^, ^65^, ^66^, ^68^, ^70^, ^71^, ^72^ ^]^

Applying an SSLP marker diagnostic for the 831‐bp TE indel at *ZmRRS1*, we genotyped 66 Huang‐Huai‐Hai elite accessions with field pest‐/disease‐resistance phenotypes (Table S8, Supporting Information). We obtained 38 (57.6%) carry the resistant *ZmRRS1* ^Hap1^ allele, 23 (34.8%) the susceptible *ZmRRS1* ^Hap2^ allele, and five (7.6%) are heterozygous. The prevalence of the TE deletion highlights this non‐coding variant as a practical breeding target for sustainable BLSB resistance.

### 3.3 Growth‐Defense Balance

Balancing growth and immunity remains a central challenge in crop improvement.^[^ ^73^ ^]^ Constitutive activation of classic immune regulators (e.g., *NPR1*, *RPM1*, *RPS5*) often triggers autoimmunity, stunting, and yield loss.^[^ ^74^, ^75^, ^76^ ^]^ By contrast, *ZmRRS1* does not raise basal ROS in the absence of pathogens; infection licenses a rapid, inducible oxidative burst that restricts *R. solani* while leaving core agronomic traits unchanged (Figure 6A‐D; Figure 7A,B; Figure S7A, Supporting Information). Consistent with a homeostatic, rather than chronic, ROS state, GO terms for “cellular detoxification” and “oxidoreductase activity” are enriched, indicating coordinated antioxidant buffering (Figure 5B,C). Mechanistically, perception modules (ZmWAKL‐ZmWIK‐ZmBLK1‐ZmRBOH4) generate transient apoplastic ROS,^[^ ^23^ ^]^ while the SA–NPR1 pro‐survival hub curbs runaway cell death and preserves redox balance.^[^ ^57^ ^]^ Field‐relevant precedents align with this context‐dependent model: rice *UMP1R^2115^* elevates H_2_O_2_ via proteasome‐mediated turnover of peroxidases/catalases without yield penalties,^[^ ^77^ ^]^ and *bsr‐d1* sustains H_2_O_2_ to confer broad‐spectrum blast resistance at no apparent growth cost.^[^ ^78^ ^]^ Conversely, constitutively high/systemic ROS in lesion‐mimic backgrounds (e.g., LSD1‐pathway defects) drives growth retardation and precocious senescence,^[^ ^79^, ^80^ ^]^ phenotypes absent in *ZmRRS1*‐OE. Together, the data support a model in which infection “licenses” *ZmRRS1* to transiently amplify early ROS for defense without growth costs, enabling deployment of the elite *ZmRRS1* ^Hap1^ allele without the yield penalties common to classical R genes; analogous growth–defense harmony has been engineered at multigenic loci (epigenetically balanced antagonistic NLR pair in rice; *IPA1*, *RBL1*, *bsr‐d1*, and wheat *TaMlo‐R32*).^[^ ^66^, ^78^, ^81^, ^82^, ^83^ ^]^

The natural 831‐bp TE indel in the *ZmRRS1* promoter is readily tracked by a single SSLP marker, facilitating rapid introgression of the resistant *ZmRRS1* ^Hap1^ allele via marker‐assisted selection. Given that the rice orthologue *OsMDH1/8.1* confers similar protection, CRISPR‐based promoter editing or transgenic stacking could extend BLSB resistance across cereals. Critically, *ZmRRS1* ^Hap1^ enhances defense without compromising growth or yield, making it an attractive target for elite hybrids. Layering *ZmRRS1* with complementary quantitative loci that fortify cell walls or hormone circuits should build a durable, multi‐tier shield against *R. solani*. More broadly, fine‐tuning the timing and amplitude of immune outputs, rather than relying on simple ON/OFF switching, can provide robust protection while maintaining productivity.

### 3.4 Limitations of the Study

Despite these advances, key gaps remain. (i) Pathogen‐regulated methylome. It is worthy to further understand how pathogens remodel the methylome to re‐programme defense genes expression on a genome‐wide level. (ii) Field durability. The longevity of *ZmRRS1*‐mediated resistance under mixed infections, abiotic stresses, and diverse germplasm, as well as any latent metabolic trade‐offs, requires validation through multi‐environment trials. Resolving these questions will be pivotal for integrating *ZmRRS1* into broader immune networks and breeding pipelines.

## 4 Experimental Section

### Plants Materials and Growth Conditions

The natural maize population used in this study was reported previously^[^ ^44^ ^]^ and was obtained from the National Maize Improvement Center of China (NMICC), China Agricultural University, Beijing. All transgenic maize lines and CRISPR‐derived loss‐of‐function mutants were generated by the Center for Functional Genomics and Molecular Breeding of Crops at China Agricultural University in Beijing in the “ND101” background, which harbors the *ZmRRS1* ^Hap2^ allele. The EMS mutant line *zmwrky44* (B73 background; MEMD ID: EMS4‐1c0b63) was obtained from the Maize EMS‐induced Mutant Database (http://elabcaas.cn/memd/). The BC_2_ recombinant inbred lines (BC_2_RILs) population used for fine‐mapping was reported by Lv et al. (2021);^[^ ^47^ ^]^ it derives from a single‐seed descent CML496 × Lx9801 cross with Lx9801 as the recurrent parent. Within this population, we identified three lines, CG2395, CG2350, and CG2453, that segregate in the *ZmRRS1* promoter.

Maize field trials were conducted at the Shang Zhuang, Beijing, China (40.13°N, 116.10°E). Wild‐type maize inbred line ND101, together with *ZmRRS1* overexpression (*ZmRRS1*‐OE) and knockout (*zmrrs1*‐KO) transgenic lines, were grown side‐by‐side in the same plot. Each genotype was sown in three to four randomly assigned rows. Seeds were planted in 3.5‐meter‐long rows, with 15 plants per row, a plant spacing of 25 cm, and a row spacing of 50 cm. Standard agronomic practices were followed, including fertilization and irrigation. The daily average temperature during the growing period was ≈28 °C, with a relative humidity of ≈70%. No fungicide treatment was applied. At the V10 stage, all plants were artificially inoculated with *R. solani*, as described below.

Maize seeds were sown in a plastic pot (9 cm × 5.5 cm × 7.5 cm, length × width × depth) and grown in a greenhouse for 14 days (V2‐V3 stage) at 25 °C under a 16‐h light/8‐h dark photoperiod with 200 µmol m^−2^s^−1^ white light and 60% relative humidity. The seedlings were then used for subsequent indoor inoculation with *R. solani*.

Transgenic rice lines overexpressing *OsMDH1* (*pUbi::OsMDH1*‐FLAG, *OsMDH1*‐OE) or *OsMDH8.1* (*pUbi::OsMDH8.1*‐FLAG, *OsMDH8.1*‐OE) in the Nipponbare background were grown in a greenhouse at 28 °C and 80 % relative humidity under a 12 h: 12 h light‐dark cycle with a photon flux density of 200 µmol m^−2^s^−1^.

### *R. solani* Inoculation and Disease Phenotype Evaluation

For field sheath inoculation of maize with *R. solani*, maize plants grown under standard field conditions were inoculated with *R. solani* at 55 days after sowing (V10 stage). The *R. solani* XN strain was revived on potato dextrose agar (PDA) at 28 °C for 3 days,^[^ ^84^ ^]^ sub‐cultured once, and grown for an additional 48 h until the colony reached the plate margin. Twelve uniform mycelial plugs (Ø = 6 mm, cut from the expanding edge with a No. 2 cork borer) were transferred to 200 mL potato dextrose broth (PDB) in a 500‐mL Erlenmeyer flask and incubated for 3.5 days (28 °C, 220 rpm) until dense, uniformly suspended hyphal pellets formed. The culture was homogenized in a household blender for 30–40 s with intermittent pauses (5–10 s) to prevent heat buildup, until a uniform mycelial suspension devoid of visible solid fragments was obtained. All homogenates were subsequently pooled and thoroughly mixed in a plastic pail in preparation for field inoculation.

Filter‐paper strips (15 × 2 cm; consistent dimensions) were thoroughly soaked in the uniform PDB‐based fungal suspension before inoculation. Each strip was uniformly affixed to the leaf sheath at the fourth internode and promptly sealed with five layers of cling film to ensure complete coverage and maintain localized humidity at the inoculation site. Inoculations were performed in a single afternoon when ambient temperatures were moderate. Drip irrigation (10–15 min) was applied immediately afterward, and the plots were misted each evening for the first 3–5 days to sustain high humidity. Elliptical grey‐green lesions appeared 3–5 dpi, later turning yellow to reddish‐brown and producing sclerotia. Lesion length was measured and photographed at 10–14 dpi, and disease indices were calculated for phenotypic scoring.

For the detached‐leaf assay in maize, V2‐V3 stage seedlings (14 days after sowing) were excised and placed abaxial side up in a growth chamber. Mycelial plugs (Ø = 6 mm, No. 2 cork borer) cut from the expanding margin of an *R. solani* PDA culture (one subculture, 48 h, 28 °C) were positioned on the leaf surface. Inoculated leaves were incubated at 28 °C under continuous high humidity. Lesion development was photographed 24–36 hpi, and the lesion area was quantified in ImageJ (v. 1.53).

For greenhouse sheath inoculation in rice, a suspension culture was prepared by transferring twenty 4‐mm plugs of *R. solani* into 200 mL PDB and shaking for 3.5 days (28 °C, 220 rpm) to yield uniform hyphal pellets. At the panicle initiation stage, one pellet was placed between the outer and inner leaf sheaths; the area was wrapped with damp cotton and sealed with aluminum foil to preserve humidity. Plants were kept in dim, humid conditions and misted daily. Lesion length was recorded and photographed at 7 dpi. Fungal biomass analysis involved pooling mRNA from 8 to 12 inoculated plants per treatment for quantitative PCR with SYBR Green (TSE401; TsingKe Biotech, Beijing) on a QuantStudio 6 Flex system (Thermo Fisher Scientific, Waltham, MA). Transcript abundance was normalised to *R. solani Actin* and expressed relative to *Oryza sativa Actin*.

### Phenotyping of Maize BLSB Resistance and GWAS

Phenotyping of BLSB resistance was conducted in a natural‐variation population of 302 maize inbred lines from a previously described association panel,^[^ ^44^ ^]^ publicly available through MaizeGo. For each genotype, 5–10 V10 plants were inoculated in vivo at the leaf sheath with *R. solani* as detailed above, and lesions were photographed and measured 14 dpi. A disease index (DI) from 0 to 7 was assigned on the basis of lesion length and visual severity (Figure 1B); DI scores for all 302 lines are listed in Table S1, Supporting Information. The frequency distribution of DI values was plotted in R (v 4.3.0) with ggplot2.

Genome‐wide association mapping was performed in TASSEL 5.0^[^ ^85^ ^]^ using 1252883 high‐quality SNPs (MAF ≥ 0.05) reported by Li et al. (2013).^[^ ^45^ ^]^ After comparing a general linear model that accounts for population structure (Q), we adopted the mixed linear model (MLM) to control for both structure and kinship. The association between each SNP genotype and phenotype was calculated using TASSEL, with a significance threshold set at *p* < 1 × 10^−5^. Manhattan plot and QQ plot were generated using the R package “CMplot,” and LD analysis of the candidate interval was visualized using the R package “LDheatmap.”

### Plasmid Constructs and Genetic Transformation

Maize transgenic plants and CRISPR‐Cas9 mutants were generated in the ND101 background by the Center for Crop Functional Genomics and Molecular Breeding at China Agricultural University, Beijing. We obtained *pUbi::ZmRRS1* overexpression lines (*ZmRRS1*‐OE1 and *ZmRRS1*‐OE2) by overexpressing the *ZmRRS1* (*GRMZM2G068455*) coding sequence (CDS) from B73 in the pBCXUN vector (XcmI). To create *ZmRRS1* knockout mutants, CRISPR‐P (http://crispr.hzau.edu.cn/CRISPR2/) was used to target the malate dehydrogenase domain of *ZmRRS1*. This was cloned into the pBUE411 vector (BsaI) and edited using CRISPR‐Cas9, resulting in *zmrrs1*‐KO1 and *zmrrs1*‐KO2 mutants.^[^ ^92^ ^]^ For *OsMDH1* and *OsMDH8.1* overexpression in rice, we cloned their CDS from Nipponbare into the pCAMBIA1300‐Ubi vector (MluI and HindIII), generating the *pUbi*::*OsMDH1*‐FLAG and *pUbi*::*OsMDH8.1*‐FLAG constructs.

To assess BLSB resistance, this work utilized the ZMBJ‐CMV VIGS system in maize B73, using vectors pCMV101, pCMV201, and pCMV301. Specific VIGS fragments (200–300 bp) were designed for genes *A1* (*GRMZM2G471876*), *A2* (*GRMZM2G068455*) and *ZmWRKY44* (*GRMZM2G432583*) according to Wang et al. (2016) (http://vigs.solgenomics.net).^[^ ^46^ ^]^ The specific amplified cDNA fragments were cloned into the pCMV201‐2b_N81_vector (KpnI and XbaI).

To generate His‐MBP‐*ZmRRS1*‐FLAG and His‐MBP‐*ZmWRKY44*‐FLAG constructs, the CDS of *ZmRRS1* (from B73 and CML496) and *ZmWRKY44* (from B73) were amplified and cloned into the pET1a His‐MBP‐FLAG vector (SfiI). The *ZmWRKY44* CDS is identical in both the B73 and CML496 backgrounds. To construct *ZmWRKY44*‐pB42AD and *W‐box*/*mW‐box*‐pLacZ2µ, the *ZmWRKY44* CDS (from B73) and the *W‐box*/*mW‐box* from the *ZmRRS1* promoter (Figure 4B) were PCR‐amplified. Notably, the W‐box sequences are conserved between B73 and CML496. The fragments were then cloned into pB42AD and pLacZ2µ using the EcoRI and XhoI sites, respectively. To construct the *pZmRRS1* ^B73^::LUC and *pZmRRS1* ^CML496^::LUC, this work amplified the 1.864 and 1.379 kb promoters of *ZmRRS1* from the B73 and CML496, respectively, and cloned them into the pGreenII0800‐LUC (HindIII and NcoI). To generate *ZmWRKY44*‐HA and *ZmWRKY44*‐GFP constructs, the *ZmWRKY44* CDS from B73 was amplified and cloned into the pCB35S‐HA and pCB35S‐GFP (SfiI). To generate *p35S::ZmRRS1* ^B73^‐GFP and *p35S::ZmRRS1* ^CML496^‐GFP constructs, the *ZmRRS1* CDSs from B73 and CML496 were amplified and cloned into the pCB35S‐GFP (SfiI). All primer and guide RNA sequences are detailed in Table S9, Supporting Information. Transgenic plants were produced using *Agrobacterium*‐mediated methods.^[^ ^86^ ^]^ All plasmid inserts and junctions were verified by Sanger sequencing.

### RNA Extraction and RT‐qPCR

Total RNAs were extracted using M5 SuperPure Total RNA Extraction Reagent (MF736‐01, Mei5 Biotechnology Co., Ltd., Beijing, China) according to the manufacturer's protocols. First‐strand cDNA was synthesized with 1 µg starting total RNA using a StarScript Pro All‐in‐one RT Mix with gDNA Remover (A240, GenStar, Beijing, China). RT‐qPCR was performed with an ArtiCan^CEO^ SYBR qPCR Mix (TSE401; TsingKe Biotech, Beijing) on a QuantStudio 6 Flex Real‐Time (Thermo Fisher Scientific, Waltham, MA). *ZmActin* and *Zm18S* were used as maize internal reference genes, whereas *OsActin* served as the housekeeping control for rice. The relative expression was calculated using the 2^‐ΔΔ^ *^C^* ^t^ method. The primers are listed in Table S9, Supporting Information. Each experiment was conducted with at least three biological replicates.

### RNA‐Seq Analysis

For RNA‐seq, 55d (V10) field‐grown *ZmRRS1*‐OE and ND101 wild‐type plants were sheath‐inoculated with *R. solani* or water (mock) as described above. Inoculated leaf sheaths were harvested 24 hpi, and total RNA was extracted with TRIzol. Libraries were prepared by Beijing Tsingke Biotechnology Co., Ltd. and sequenced on an Illumina NovaSeq 6000 platform.

Adapter and low‐quality bases were trimmed with fastp (v0.20.1, default settings). Clean reads were aligned to the maize reference genome (B73 RefGen_v4, AGPv4) using HISAT2 (v2.0.4); data quality was assessed with FastQC (v0.11.9). Gene‐level counts were obtained with featureCounts (v2.0.1). Eight cDNA libraries generated 14.91 b, 13.58 b, 14.87 b, and 14.24 b high‐quality clean reads from the OE‐Mock, OE‐*R. solani*, WT‐Mock, and WT‐*R. solani* samples, respectively (Table S10, Supporting Information); 94.95–95.36 % of these reads aligned to the maize B73 RefGen_v4 genome (Table S11, Supporting Information), attesting to the robustness and reproducibility of the dataset. DEGs were defined at *p* < 0.05 and |log_2_(fold change)| ≥ 1.5 (*ZmRRS1*‐OE versus wild type). DEG clustering was visualized with the R package pheatmap (v1.0.12), and GO enrichment was performed with agriGO v2 using an FDR threshold of 0.01.^[^ ^87^ ^]^

### Assay of MDH Activity

Malate dehydrogenase activity was quantified by monitoring the oxidation of NADH to NAD^+^ (ΔA_340_). Recombinant ZmRRS1^B73^ and ZmRRS1^CML496^ proteins, expressed in *Escherichia coli* BL21 (DE3) and affinity‐purified, were assayed alongside an empty‐vector FLAG control. Aliquots of 20 µg purified protein were tested with the NAD‐MDH kit (NMDH‐2‐Y; Suzhou Keming Biotech). Immediately after substrate addition, absorbance at 340 nm was recorded at 20 s and 80 s on a UV–vis spectrophotometer, and activities (nmol min^−1^ mg^−1^) were calculated from the ΔA_340_ using the kit's formula.

### Subcellular Localization

To assess the subcellular distribution of ZmRRS1, mesophyll protoplasts were isolated from B73 and CML496 leaves as described by Liu et al. (2024).^[^ ^88^ ^]^ Protoplasts were transfected with *p35S::ZmRRS1* ^B73^‐GFP or *p35S::ZmRRS1* ^CML496^‐GFP; the empty vector pJC576‐35S‐GFP served as a control. After a 15–18 h incubation at 25 °C in darkness, GFP and mCherry signals were visualized with a Carl Zeiss LSM 800 confocal microscope (excitation 488 and 561 nm, respectively).

### Phylogenetic Analysis

The ZmRRS1 amino acid sequence was downloaded from MaizeGDB (https://www.maizegdb.org/). Orthologs and paralogs were identified, and their phylogenetic relationships visualized using OrthoVenn3 (https://orthovenn3.bioinfotoolkits.net).^[^ ^89^ ^]^ By entering the protein ID Zm00001eb179730 in the “Orthologs analysis” module, the ZmRRS1 phylogenetic tree was automatically generated and exported for further analysis.

### Virus Induced Gene Silencing

The CMV‐based VIGS assay was performed essentially as described by Wang et al. (2016).^[^ ^46^ ^]^ The pCMV201‐2b_N81_ construct and the helper plasmids pCMV101 and pCMV301 were electroporated into *Agrobacterium tumefaciens* strain C58C1. Equal volumes of the three cultures (1:1:1) were mixed and pressure‐infiltrated into fully expanded *Nicotiana benthamiana* leaves for transient expression. Four days after infiltration, the infiltrated tissue was harvested, homogenized in a 10 mM phosphate buffer (pH 7.0), and centrifuged at 4 °C (4500 g, 3 min). The resulting crude sap, containing virus, was used to inoculate maize (B73) kernels by the vascular‐puncture method.^[^ ^46^ ^]^ Inoculated seeds were kept in the dark at 25 °C for 2 days, then transferred to 10‐cm pots and grown under a 16 h light (20 °C) / 8 h dark (18 °C) photoperiod. Seven days after sowing, seedlings exhibiting a mosaic phenotype were evaluated for silencing efficiency; plants with a reduction of 50% or more in target transcript abundance were moved to the V4 stage for subsequent inoculation.

### Yeast One Hybrid Assay

ZmWRKY44 fused to the pB42AD activation domain was co‐transformed into *Saccharomyces cerevisiae* strain EGY48 with either the W‐box‐pLacZ2µ or mW‐box‐pLacZ2µ reporter; empty vectors (pB42AD + pLacZi) served as negative controls. Transformants were selected on SD/‐Ura/‐Trp medium containing 2 % glucose, then transferred to induction plates (SD/‐Ura/‐Trp, 2 % galactose, 1 % raffinose) supplemented with 80 µg mL^−1^ X‐Gal to assess ZmWRKY44 binding to the *ZmRRS1* promoter W‐box motif. Primer sequences are provided in Table S9,Supporting Information.

### Electrophoretic Mobility Shift Assay

EMSA was performed as described by Li et al. (2022).^[^ ^90^ ^]^ Recombinant MBP‐ZmWRKY44‐FLAG protein was incubated at 25 °C for 20 min with biotin‐labelled, competitor, or mutated probes using a chemiluminescent EMSA kit (GS009, Beyotime, Shanghai). Reactions were halted using a 10 × native loading buffer, resolved on a native‐PAGE gel, and detected by chemiluminescence. The DNA sequences of the probes are listed in Table S9, Supporting Information.

### Transcriptional Activity Assay

Dual‐luciferase (LUC) assays were performed essentially as described by Li et al. (2022).^[^ ^90^ ^]^ Mesophyll protoplasts isolated from 14d etiolated maize seedlings were co‐transfected with the vector *p35S::ZmWRKY44*‐HA and either *pZmRRS1* ^B73^ *::LUC* or *pZmRRS1* ^CML496^ *::LUC* reporter constructs at 25 °C for 12 h. For the Chitin treatment, samples were treated with a final concentration of 200 µg mL^−1^ and the suspension was gently shaken (55 rpm, 10 min). The *p35S::REN* was used as an internal control. Firefly luciferase (LUC) and Renilla luciferase (REN) activities were measured with a commercial substrate kit (Promega) on a multimode plate reader (F200, Tecan, Switzerland). Promoter activity was quantified as the LUC/REN ratio.

### Chromatin Immunoprecipitation‐qPCR Assay

Chromatin immunoprecipitation (ChIP) was performed with minor modifications to the protocol of Kaufmann et al. (2010).^[^ ^91^ ^]^ High‐quality mesophyll protoplasts (5 × 10^−5^ cells mL^−1^) were isolated from 14d etiolated CG2395 BB and CG2395 CB seedlings. For each genotype, 6 mL of protoplast suspension was transfected with 500 µg of *p35S::ZmWRKY44*‐GFP plasmid and incubated for 12 h at 25 °C in the dark. Chitin‐treated samples received 200 µg mL^−1^ chitin (55 rpm, 10 min), while water‐treated protoplasts served as controls.

Cells were cross‐linked with 1% formaldehyde for 10 min at 25 °C, and the reaction was quenched by adding 2 M glycine to a final concentration of 0.125 M (5 min at room temperature, followed by 3 min on ice). Nuclei were isolated, chromatin was extracted and sonicated into 200–300 bp fragments, then immunoprecipitated with Protein A agarose beads (Santa Cruz, sc‐2001) pre‐bound to mouse anti‐GFP monoclonal antibody (AE012, ABclonal). Antibody specificity for ZmWRKY44‐GFP was validated by anti‐GFP immunoblotting in CG2395 BB and CB protoplasts. Immuno‐enriched DNA was quantified by qPCR using the primers listed in Table S9, Supporting Information. Fold enrichment was calculated as the ratio of the percentage of input obtained with anti‐GFP compared to nonspecific IgG, where the percentage of input is equal to 2^(Ct input‐Ct IP)^ × 100 %.

### Bisulfite Sequencing

For bisulfite sequencing, leaf sheaths of CG2395BB and CG2395CB plants were collected at 24 hpi with *R. solani*; tissues at 0 h served as controls. Genomic DNA was isolated with the Hi‐DNAsecure Plant Kit (DP350, Tiangen, Beijing). DNA was fragmented to ≈350 bp using the S‐TN5 transposase complex Mix (TDE0506, Tsingke), and libraries were prepared with the S‐1.2×‐TN5 Library Enrichment Mix (TDE0505, Tsingke). After adaptor ligation, bisulfite conversion was performed with the EpiArt Ultrafast DNA Methylation Bisulfite Kit (EM112, Vazyme) according to the manufacturer's instructions, and target promoter regions were enriched by nested PCR using 2× Tsingke Master Mix (blue) (TSE004). Indexed libraries were pooled and sequenced on an Illumina MiSeq with the MiSeq Reagent Kit v2 (300‐cycles; MS‐102‐2002) to generate 2×150 bp paired‐end reads.

Raw reads were adapter‐trimmed and quality‐filtered with fastp v0.20.1 (default settings; minimum read length 50 bp). For locus confirmation and variant screening, sequences were assembled with SPAdes v3.14.1. Reads were aligned to the reference genome with bwa v0.7.17 and processed with samtools v1.10 to produce coordinate‐sorted BAMs. SNPs within the targeted interval were identified with snippy v4.6.0. Base composition (A/C/G/T) at each reference position was summarized from BAMs using bam‐readcount; per‐site methylation frequency was computed as C/(C+T) on the reference forward strand (and G/(G+A) on the reverse complement). Unless otherwise stated, default parameters were used.

### Detection of Reactive Oxygen Species

For 3,3′‐diaminobenzidine (DAB) staining, maize leaves harvested 36 h after *R. solani* inoculation were immersed overnight at 25 °C in a 1 mg mL^−1^ solution of DAB (pH 3.8); leaves collected at 0 h served as controls. Samples were destained with a mixture of ethanol, acetic acid, and glycerol (3:1:1, v/v/v), and the reddish‐brown precipitate indicating H_2_O_2_ accumulation was imaged using a Nikon Ti‐E fluorescence microscope.

For quantitative H_2_O_2_ determination, 0.1 g of fresh leaf tissue (36 h post‐inoculation or 0 h control) was ground in 1 mL of ice‐cold acetone, and the homogenate was centrifuged at 8000 × g for 10 min at 4 °C. The supernatant was assayed using a Hydrogen Peroxide Assay Kit (BC3595; Solarbio, Beijing, China) according to the manufacturer's protocol.

### H_2_O_2_/Mitochondria Co‐Staining and Confocal Microscopy

Leaf tissue from *ZmRRS1*‐OE plants inoculated with *R. solani* was sampled at 24 hpi. Segments were pretreated with 200 mM NaCl for 30 min and then incubated in staining solution containing 100 nM MitoTracker Red CMXRos (Beyotime, C1049B) prepared in 1× HBSS with Ca^2+^/Mg^2+^ (Biotopped, Top0049S), protected from light. Samples were vacuum‐infiltrated twice (2 min each; avoid prolonged vacuum). The staining solution was then kept in a light‐protected water bath at 37 °C for 40 min. H_2_O_2_ was labeled by adding H_2_DCFDA to a final concentration of 10 µM (also known as DCFH‐DA; 2′,7′‐dichlorodihydrofluorescein diacetate; MedChemExpress, CAS 4091‐99‐0) and incubating in a light‐protected water bath at 37 °C for 20 min. Before imaging, tissues were rinsed twice in 10 mM PBS (5 min each).

Fluorescence images were acquired on a TCS SP8 confocal microscope (Leica Microsystems). Detector settings were: H_2_DCFDA, excitation 488 nm and emission 517–527 nm; MitoTracker Red, excitation 552 nm and emission 560–615 nm.

### Determination of Yield‐Related Traits

Field evaluations were conducted in summer 2024 at the Shang Zhuang, Beijing, China (40.13° N, 116.10° E), using wild‐type ND101 and transgenic *ZmRRS1* overexpression (*ZmRRS1*‐OE) and knockout (*zmrrs1*‐KO) lines. A randomized complete‐block design with three replicates per genotype was employed. Each replicate comprised a two‐row plot (row length = 3.5 m; 15 plants per row) spaced 0.25 m within rows and 0.5 m between rows. Seeds were sown on 1 May 2024 and harvested on 30 September 2024. Plant height and ear height were recorded immediately before harvest; ears were subsequently dried and evaluated for ear weight, ear length, ear diameter, row number, kernels per row, 100‐kernel weight, and the mean length and width of ten representative kernels.

### Quantification and Statistical Analysis

Statistical analyses were performed using GraphPad Prism v8.0. Differences between the two groups were evaluated using two‐tailed Student's *t*‐tests. For multiple comparisons, one‐way ANOVA followed by Tukey's test or two‐way ANOVA with Tukey's test was applied. Bars marked with different letters or asterisks indicate statistically significant differences at *p* < 0.05. Additional details are provided in the corresponding figure legends.

## Conflict of Interest

The authors declare no conflict of interest.

## Author Contributions

Conceptualization: L.W., and W.Z. Methodology: L.W., J.C., C.L., D.L., M.D., S.H., W.C. and W.Z. Formal analysis: L.W. Investigation: L.W., J.C., C.L., D.L., M.D., S.H. and W.C. Writing—original draft: L.W. Writing—review, and editing: L.W., J.C., V.B., W.Z. Supervision: W.Z. Project administration: W.Z. Funding acquisition: W.Z.

## Supporting information



Supporting Information

Supporting Information

## Data Availability

All data and materials required to reproduce this work are available in this manuscript. The RNA‐seq raw reads have been deposited in the NCBI Sequence Read Archive (SRA) under BioProject PRJNA1321191 (BioSample SAMN51201532).
