# Supplementary material for: Allelic Variation in Maize Malate Dehydrogenase 7 Shapes Promoter Methylation and Banded Leaf and Sheath Blight Resistance
Source: Adv Sci (Weinh). 2025 Nov 20;13(7):e11356. doi: 10.1002/advs.202511356 (PMC12866847; doi:10.1002/advs.202511356)
Supplement: Supplementary file 1 — Supporting Information [file ADVS-13-e11356-s002.docx]

Supporting Information

Allelic variation in maize *Malate Dehydrogenase 7* shapes promoter methylation and banded leaf and sheath blight resistance

*Luyang Wei, Junbin Chen, Chuang Liu, DanDan Liu, Meida Du, Shengfeng He, Wenyu Cheng, Vijai Bhadauria, You-Liang Peng, Wangsheng Zhu**

**List of Supplementary Information**

**Figure S1.** GWAS analysis of maize resistance to BLSB.

**Figure S2.** *ZmRRS1* and its homologs positively regulate resistance to *R. solani* in maize and rice.

**Figure S3.** A natural 831-bp indel regulates expression of *ZmRRS1* and BLSB resistance.

**Figure S4.** *ZmWRKY44* regulates resistance to *R. solani* in maize.

**Figure S5.** *ZmWRKY44* acts upstream of *ZmRRS1* to promote resistance to *R. solani*.

**Figure S6.** Transcriptomic analysis of *ZmRRS1*-regulated differentially expressed genes (DEGs).

**Figure S7.** *ZmRRS1* activates ROS-SA signaling.

**Figure S8.** Yield-related traits of *ZmRRS1* transgenic lines under normal conditions.

**Table S1.** List of the 302 varieties used in this study.

**Table S2.** List of candidate genes identified through GWAS for BLSB resistance in maize.

**Table S3.** Information on 26 SNPs and the 831-bp insertion–deletion (indel) status within the *ZmRRS1* genomic region across 276 maize inbred lines.

**Table S4.** The cis-acting elements in the *ZmRRS1* promoter.

**Table S5.** Maize orthologs of resistance-associated WRKY hub genes from the *R. solani*-responsive defense GRN of *Brachypodium distachyon*.

**Table S6.** Genes differentially expressed in *ZmRRS1*-OE lines compared with the ND101 plants at non-inoculated conditions (*ZmRRS1*-OE-Mock vs ND101-Mock).

**Table S7.** Genes differentially expressed in *ZmRRS1*-OE lines compared with the ND101 plants under 24h-*R.s.* conditions (*ZmRRS1*-OE-24h-*R.s.* vs ND101-24h-*R.s.*).

**Table S8.** Genotyping of 66 disease- and pest-resistant elite germplasm accessions from the Huang–Huai–Hai region using the 831-bp SSR marker.

**Table S9.** List of the primers used in this study.

**Table S10.** Overview of RNA-seq data.

**Table S11.** RNA Sequencing data and reference genome alignment results.

**Note:** Supplementary Tables S1-S11 are provided as separate files.

**
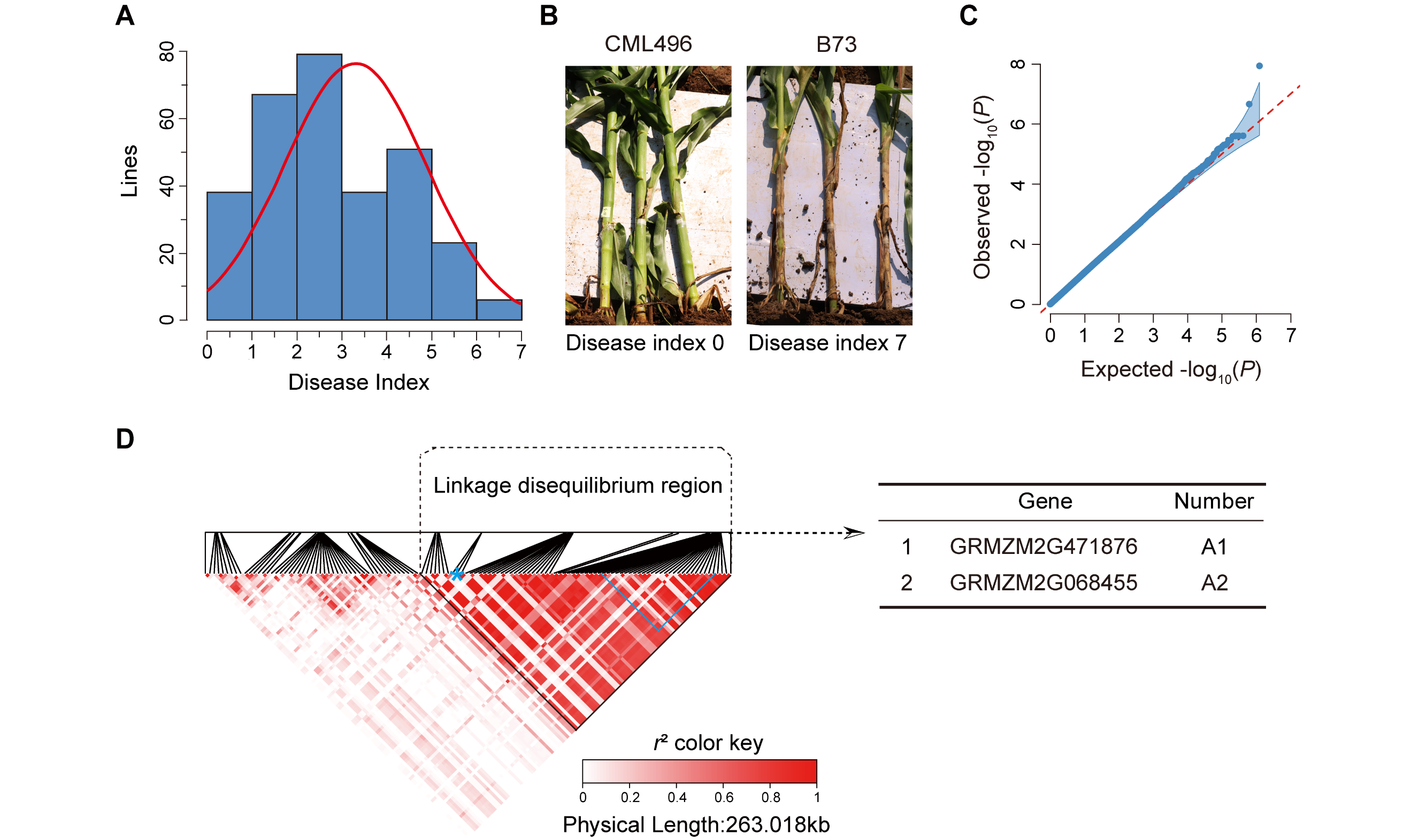
**

**Figure S1. GWAS analysis of maize resistance to BLSB.**

**(A)** Distribution of BLSB disease index in leaf sheaths of 302 maize inbred lines after *R. solani* inoculation.

**(B)** Representative disease index 0 (CML496) and 7 (B73).

**(C)** Quantile–quantile plot for GWAS under a mixed linear model (MLM).

**(D)** Linkage-disequilibrium (LD) landscape flanking the peak SNP on maize chromosome 4. Left: An LD heatmap spanning a 263.0 kb window (chr4: 83,250,460–83,513,478; 120 SNPs) centred on the top association signal, SNP chr4.S_83,388,487 (blue asterisk; *P* = 7.48 × 10⁻⁶, MaizeGDB v2). A solid black inverted triangle demarcates a 148.1 kb LD block (chr4: 83,365,391–83,513,478) in which markers exhibit strong linkage disequilibrium (“LD region”). This block harbors two candidate genes (*A1* and *A2*); gene *A2* lies within the interval highlighted by the blue inverted triangle (chr4: 83,505,796–83,508,031). Right: Two high-confidence candidate genes, annotated in MaizeGDB v5, reside within the 148.1 kb LD block; their corresponding gene IDs are listed in the “gene” column.

**
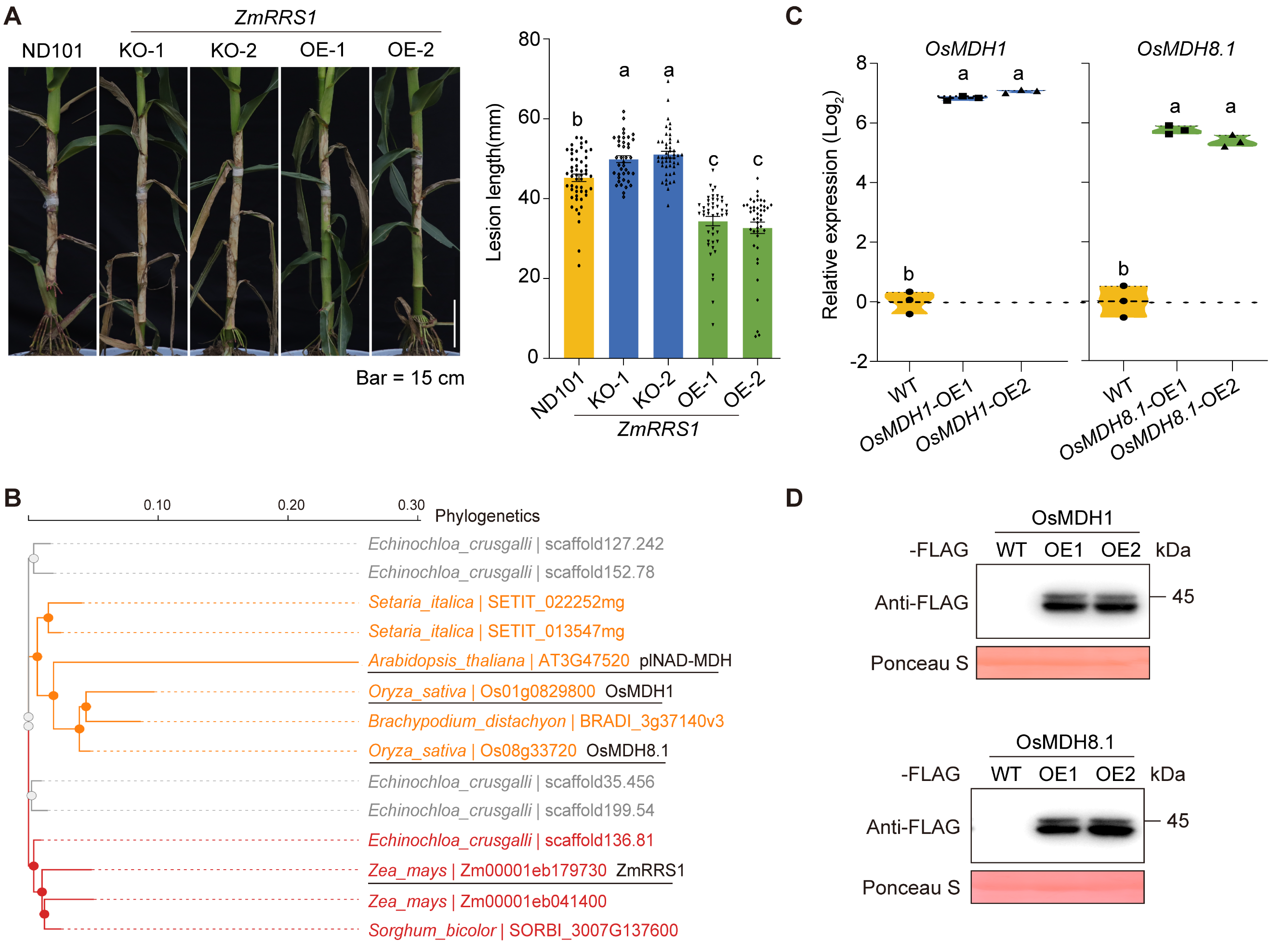
**

**Figure S2. *ZmRRS1* and its homologs positively regulate resistance to *R. solani* in maize and rice.**

**(A)** Whole-plant field phenotypes of leaf sheaths from wild type (WT), CRISPR/Cas9 knockout (*zmrrs1*-KO1 and KO2), and overexpression lines (*ZmRRS1*-OE1 and OE2) at 14 dpi. Scale bar, 15 cm. Data are shown as means ± s.e.m., *n* = 40–52 plants. The different lowercase letters indicate statistically significant differences (one-way ANOVA followed by Tukey’s test, *p <* 0.05).

**(B)** Phylogenetic analysis of ZmRRS1 and its homologs. The amino acid sequence of *ZmRRS1* was retrieved from MaizeGDB (https://www.maizegdb.org/). Orthologous and paralogous proteins were identified using OrthoVenn3 (https://orthovenn3.bioinfotoolkits.net) and used to construct the phylogenetic tree. Two ZmRRS1 orthologs were identified in *Oryza sativa* (OsMDH1 and OsMDH8.1), and one in *Arabidopsis thaliana* (plNAD-MDH). These orthologs are indicated by black horizontal bars.

**(C)** Expression levels of *OsMDH1* and *OsMDH8.1* in WT and OE lines, determined by RT–qPCR. Data are presented as means ± s.e.m., *n* = 3. Different lowercase letters indicate statistically significant differences (one-way ANOVA followed by Tukey’s test, *p* < 0.05).

**(D)** Immunoblot analysis of OsMDH1-FLAG and OsMDH8.1-FLAG protein accumulation in two independent overexpression lines in the Nipponbare background.


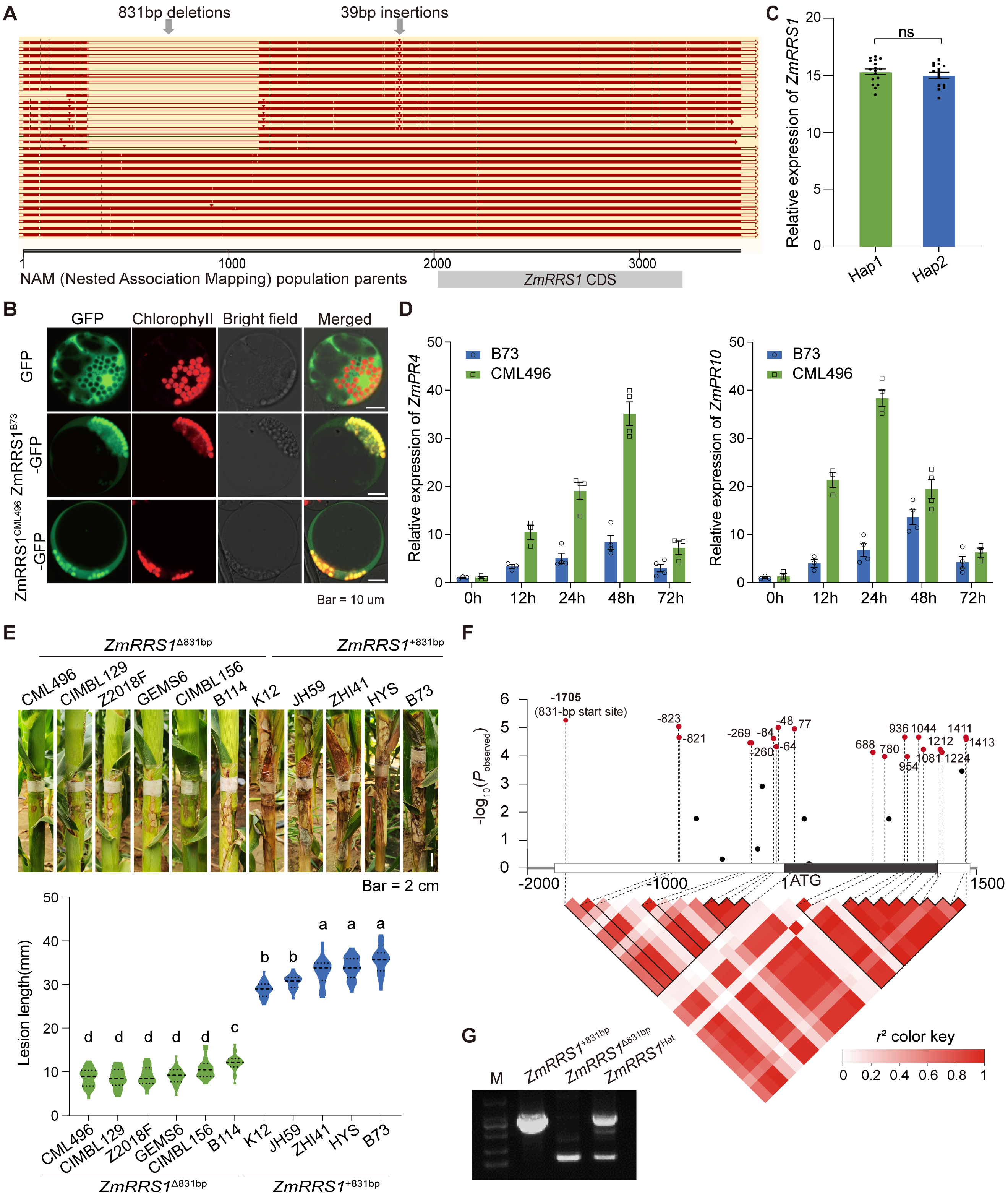


**Figure S3. A natural 831-bp indel regulates expression of *ZmRRS1* and BLSB resistance.**

**(A)** Sequence alignment illustrating natural variation in the *ZmRRS1* locus among maize NAM population parents. Grey arrows indicate two naturally occurring promoter polymorphisms—an 831-bp deletion and a 39-bp insertion, while the grey rectangle denotes the *ZmRRS1* coding region.

**(B)** Subcellular localization of ZmRRS1^CML496^-GFP and ZmRRS1^B73^-GFP in maize protoplasts. GFP fusions of ZmRRS1 from CML496 (ZmRRS1^Hap1^) and B73 (ZmRRS1^Hap2^) were transiently expressed in maize protoplasts; an empty GFP vector served as a control. Scale bar, 10 μm.

**(C)** Constitutive expression of *ZmRRS1* in resistant Hap1 versus susceptible Hap2 maize lines. Transcript levels were measured by RT–qPCR in six Hap1 and five Hap2 inbred lines. Data are presented as means ± s.e.m. (*n* = 3-6 plants per line). Statistical significance was determined by a two-tailed Student’s t-test (ns, not significant).

**(D)** Comparison of inducible *ZmPR4* and *ZmPR10* expression between the B73 (susceptible Hap2) and the CML496 (resistant Hap1). Transcript levels were quantified by RT-qPCR in leaf sheaths at 0, 12, 24, 48, and 72 hpi with *R. solani*. Data are shown as means ± s.e.m., *n* = 3-4.

**(E)** Association between the *ZmRRS1* 831-bp promoter indel and lesion length in maize inbred lines infected with *R. solani*. Top: Representative sheath symptoms 14 dpi in lines carrying the deletion allele (*ZmRRS1*^Δ831bp^, RH) versus the insertion allele (*ZmRRS1*^+831bp^, SH). Scale bar, 2 cm. Bottom: Violin plots of lesion length at 14 dpi for six *ZmRRS1*^Δ831bp^ lines and five *ZmRRS1*^+831bp^ lines (16–25 plants per line). The central line denotes the median; the outline reflects the kernel-density distribution. Different lowercase letters indicate significant differences (one-way ANOVA followed by Tukey’s test, *p* < 0.05).

**(F)** The pairwise Linkage disequilibrium (LD) matrix is derived from 26 SNPs and the 831-bp indel within the promoter and coding regions of *ZmRRS1* across 302 maize accessions. The 18 major SNPs and the 831-bp indel are highlighted in red. SNPs in strong LD with the lead SNP define LD blocks, outlined by solid lines and inverted triangles.

**(G)** The PCR-based simple sequence length polymorphism (SSLP) marker was developed to detect the 831-bp insertion/deletion. Gel electrophoresis resolved three genotypic classes: *ZmRRS1*^Δ831bp^, *ZmRRS1*^+831bp^, and *ZmRRS1*^Het^.


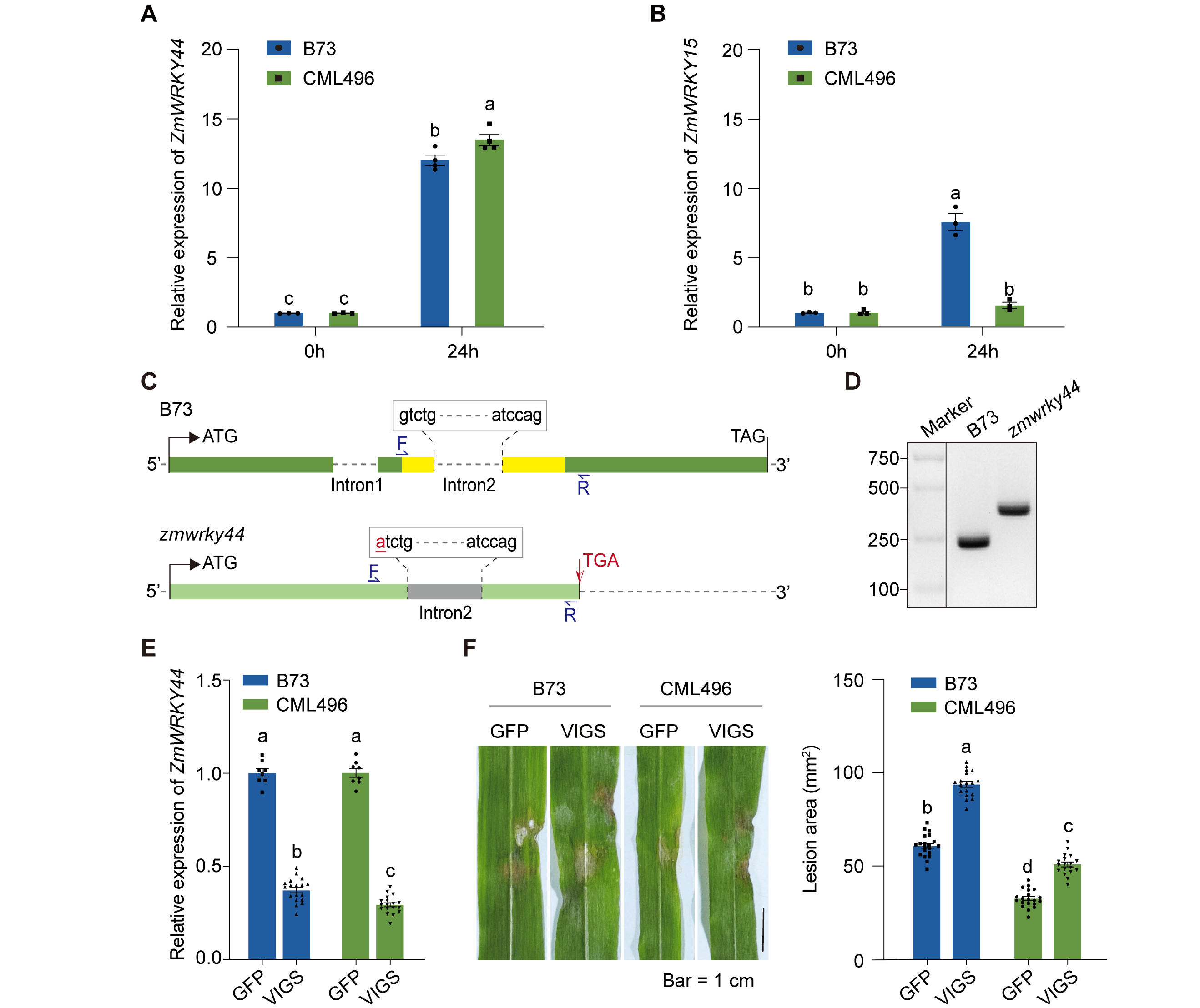


**Figure S4. *ZmWRKY44* regulates resistance to *R. solani* in maize.**

**(A-B)** RT-qPCR of *ZmWRKY44* and *ZmWRKY15* in leaf sheaths of resistant CML496 and susceptible B73 at 0 and 24 hpi (*n* = 3-4).

**(C)** Schematic of *ZmWRKY44* and the EMS-induced mutant *zmwrky44*. A 5′-GT→AT change at the intron-2 splice donor enforces intron retention and introduces a premature stop within the WRKY DNA-binding domain. The mutated base and the premature termination site are highlighted in red; exons, green boxes; retained intronic sequence arising from mis-splicing, gray boxes; WRKY DNA-binding domain, yellow box; introns, dashed lines; primer sites are marked F and R.

**(D)** RT-PCR across intron 2 reveals aberrant splicing in *zmwrky44*. Primers flanking intron 2 yield a 246-bp product in wild-type B73 and a 380-bp product in *zmwrky44* (intron retention).

**(E)** Silencing efficiency of *ZmWRKY44* assessed by RT-qPCR at 7 d after virus infiltration in B73 and CML496. Plants infiltrated with the empty vector pCMV201-2b_N81_-GFP served as controls. Data are presented as means ± s.e.m., *n* = 8-18 leaves.

**(F)** Representative phenotype (left) and lesion area quantification (right) of the second true leaf from VIGS–*ZmWRKY44* plants in B73 and CML496 at 14 d post-silencing (V2 stage), assessed 36 hours after *R. solani* inoculation. Scale bar, 1 cm. Data are presented as means ± s.e.m., *n* = 18–20 leaves.

In **(A)**, **(B)**, **(E)**, and **(F)**, Different lowercase letters indicate statistically significant differences (two-way ANOVA followed by Tukey’s test, *p* < 0.05).


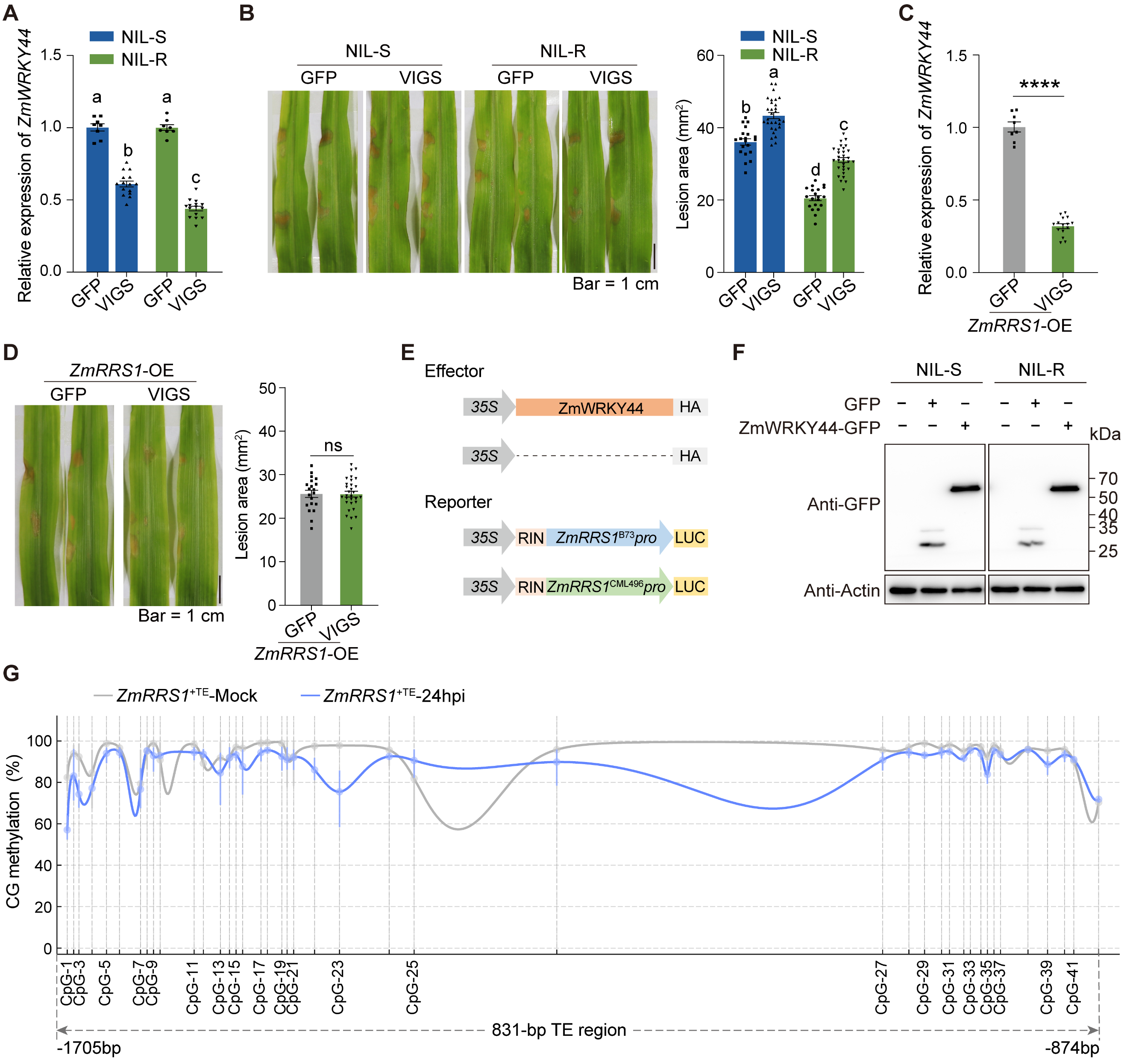


**Figure S5. *ZmWRKY44* acts upstream of *ZmRRS1* to promote resistance to *R. solani*.**

**(A and C)** Silencing efficiency of *ZmWRKY44* assessed by RT-qPCR at 7 d after infiltration in NIL-R and NIL-S (**A**), and *ZmRRS1*-OE (**C**). Plants infiltrated with the empty vector pCMV201-2b_N81_-GFP served as controls. Data are presented as means ± s.e.m., *n* = 8-15.

**(B and D)** Representative phenotype (left) and lesion area quantification (right) of the second true leaf from VIGS-*ZmWRKY44* plants in NIL-R and NIL-S **(B)**, and *ZmRRS1*-OE (**D**) at 14 d post-silencing (V2 stage), assessed 36 hours after *R. solani* inoculation. Scale bar, 1 cm. Data are presented as means ± s.e.m., *n* = 10-15.

**(E)** Schematic of plasmid constructs for the transient dual-luciferase (LUC) assay; the empty vector (EV-HA) served as the negative control.

**(F)** Protein expression validation by western blot. Protoplasts were transformed with no plasmid, empty vector (GFP), or ZmWRKY44-GFP. Total proteins were immunoblotted with anti-GFP; anti-ACTIN served as a loading control.

**(G)** CG methylation dynamics within the 831-bp TE region of the *ZmRRS1* promoter in the *ZmRRS1*^+TE^ haplotype at 0 and 24 hpi. The x-axis shows physical position relative to the ATG start codon across the 831-bp TE segment; 42 CpG sites are annotated (*n* = 3).

In **(A–C)**, different lowercase letters denote significant differences (two-way ANOVA followed by Tukey’s test, *p* < 0.05). In **(D–E)**, statistical significance was evaluated by a two-tailed Student’s *t*-test (*****p* < 0.0001, ns, not significant).


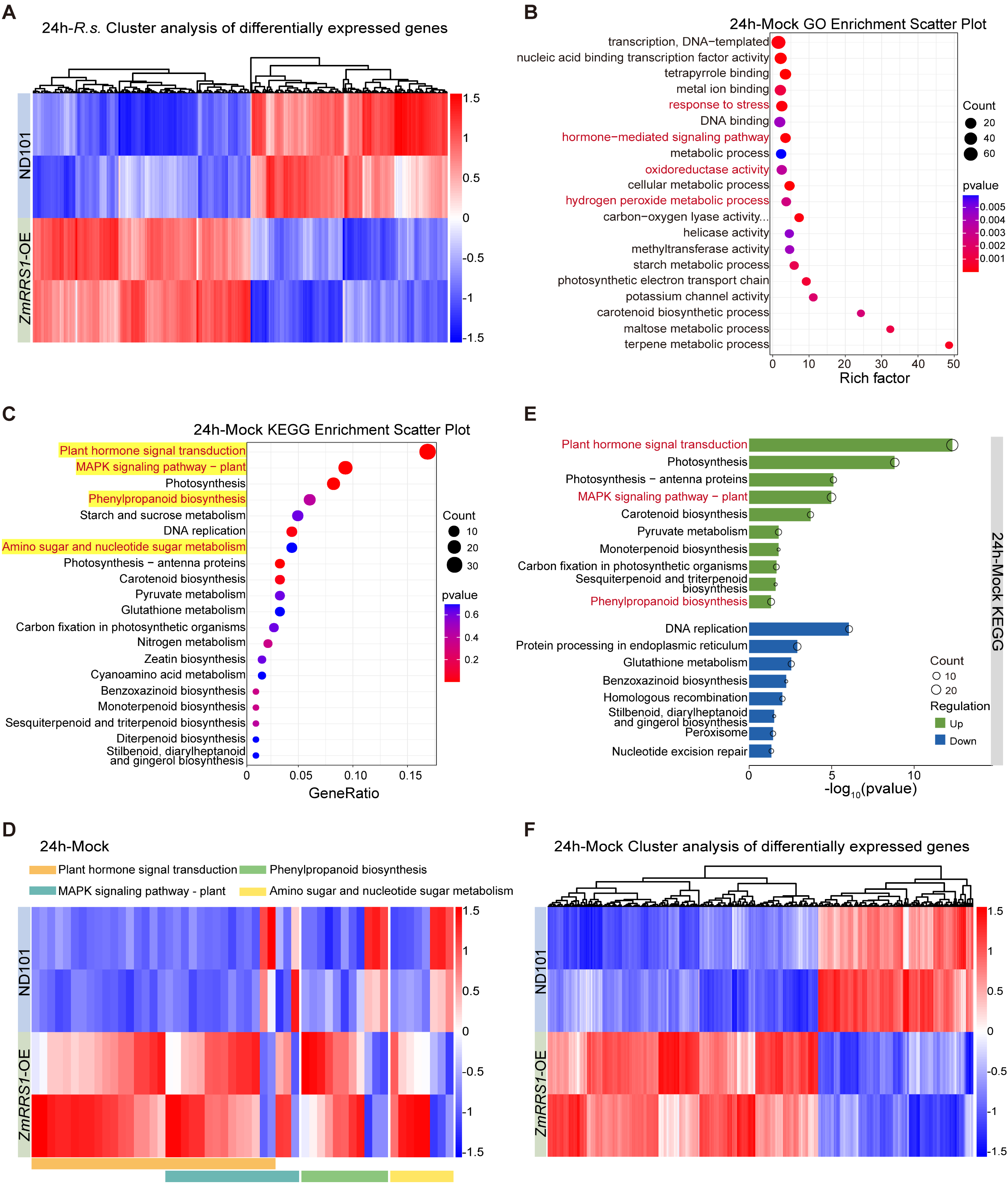


**Figure S6. Transcriptomic analysis of *ZmRRS1*-regulated differentially expressed genes (DEGs).**

**(A)** Hierarchical clustering of DEGs in ND101 and *ZmRRS1*-OE plants at 24 hpi with *R. solani*.

**(B-C)** GO and KEGG enrichment of *ZmRRS1*-dependent DEGs in mock-treated plants at 24 h. The bubble plot displays the top 20 GO terms and KEGG pathways ranked by adjusted *P*-value (*p*_adj_). The x-axis represents the rich factor and enrichment ratio, respectively, while the y-axis lists Gene Ontology (GO) terms or KEGG pathways. Bubble size represents the number of DEGs annotated per term or pathway; color reflects *p*_adj_ values. GO terms and KEGG pathways most relevant to this study are highlighted in red and yellow, respectively.

**(D)** Cluster heatmap of DEGs involved in plant disease resistance–related pathways in mock-treated plants at 24 h. Red and blue indicate high and low expression levels, respectively.

**(E)** KEGG enrichment analysis of up- and down-regulated *ZmRRS1*-dependent DEGs in mock-treated plants at 24 h. The bar chart ranks the top 18 KEGG pathways by *p*_adj_. Bars are color-coded by direction of regulation (green, up-regulated; blue, down-regulated). Pathways most relevant to this study are highlighted in red.

**(F)** Hierarchical clustering of DEGs in ND101 and *ZmRRS1*-OE plants in mock-treated conditions at 24 h.


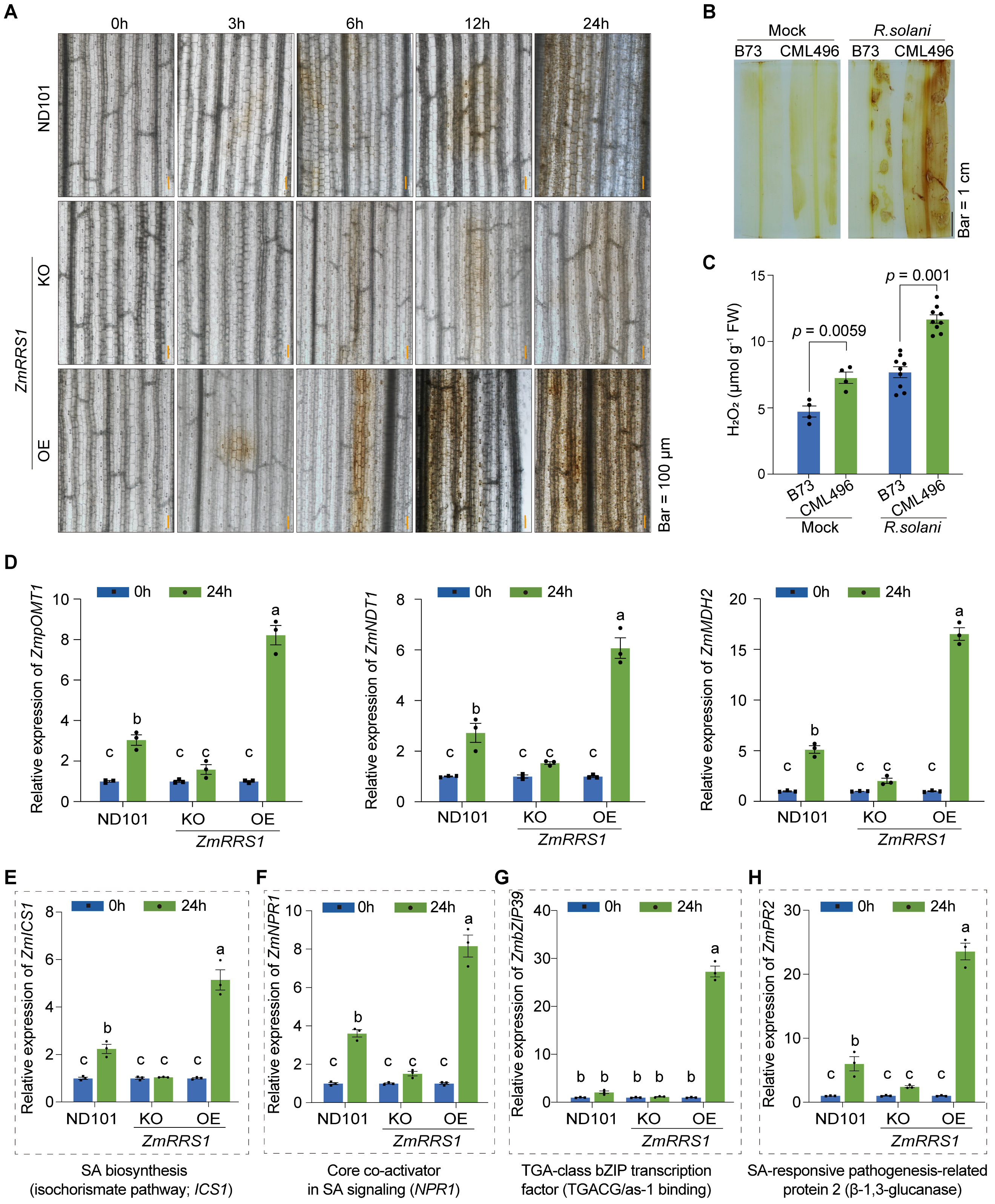


**Figure S7. *ZmRRS1* activates ROS-SA signaling.**

**(A)** DAB staining of leaves at 36 hpi with *R. solani*, showing H_2_O_2_ accumulation as dark-brown precipitates; water-treated plants served as mock controls. Scale bar, 1 cm.

**(B-C)** DAB staining and H_2_O_2_ quantification in B73 and CML496 at 36 hpi. Water-treated plants served as mock controls (*n* = 4-9). Data are presented as means ± s.e.m. Statistical significance was determined by two-way ANOVA followed by Tukey’s multiple comparisons test (*p* < 0.05).

**(D, E-H)** RT-qPCR of *ZmZmpOMT1*, *ZmMDH2*, and *ZmNDT1* (**D**), *ZmICS1* (**E**), *ZmNPR1* (**F**), *ZmZIP39* (**G**), and *ZmPR2* (**H**) in leaf sheaths of ND101, *zmrrs1*-KO, *ZmRRS1*-OE plants with *R. solani* at 0 and 24 hpi. Data are presented as means ± s.e.m., *n* = 3. Statistical significance was determined by two-way ANOVA followed by Tukey’s multiple comparisons test (*p* < 0.05).


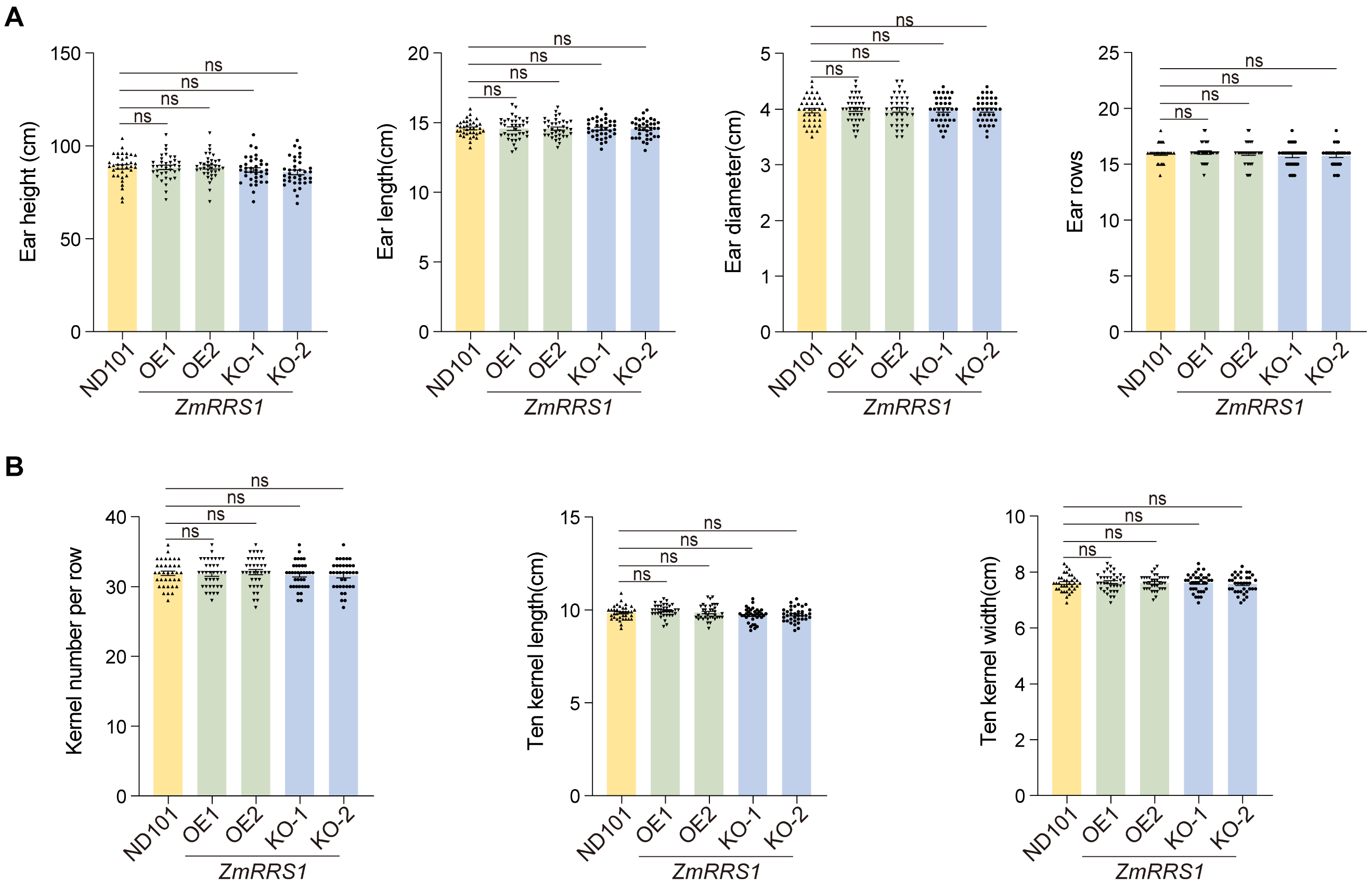


**Figure S8. Yield-related traits of *ZmRRS1* transgenic lines under normal conditions.**

**(A)** Quantification of ear height, ear length, ear width, and ear row number in *ZmRRS1* knockout (KO) and overexpression (OE) lines compared with wild type (WT) plants (*n* = 36).

**(B)** Quantification of kernels per row and mean length and width of ten kernels in *ZmRRS1*-KO and *ZmRRS1*-OE lines versus WT plants (*n* = 36).

In (A) and (B), data are presented as means ± s.e.m. Statistical significance was determined by one-way ANOVA followed by Tukey’s test (*p* < 0.05; ns, not significant).
